# Chemical Constituents, Biological Activities, and Proposed Biosynthetic Pathways of Steroidal Saponins from Healthy Nutritious Vegetable—*Allium*

**DOI:** 10.3390/nu15092233

**Published:** 2023-05-08

**Authors:** Huaxiang Wang, Qi Zheng, Aijun Dong, Junchi Wang, Jianyong Si

**Affiliations:** 1 Institute of Medicinal Plant Development, Chinese Academy of Medical Sciences and Peking Union Medical College, Beijing 100193, China; wanghuaxiang@implad.ac.cn (H.W.); zhengqi@implad.ac.cn (Q.Z.); dongaijun@implad.ac.cn (A.D.); 2 Key Laboratory of Bioactive Substances and Resource Utilization of Chinese Herbal Medicine, Ministry of Education, Beijing 100193, China; 3 Beijing Key Laboratory of Innovative Drug Discovery of Traditional Chinese Medicine (Natural Medicine) and Translational Medicine, Beijing 100193, China

**Keywords:** *Allium*, nutritious vegetable, health benefits, steroidal saponins, biological activity

## Abstract

*Allium* is a common functional vegetable with edible and medicinal value. *Allium* plants have a special spicy taste, so they are often used as food and seasoning in people’s diets. As a functional food, *Allium* also has abundant biological activities, some of which are used as drugs to treat diseases. By consuming *Allium* on a daily basis, people can receive active compounds of natural origin, thereby improving their health status and reducing the likelihood of disease. Steroidal saponins are important secondary metabolites of *Allium*, which are formed by the steroidal aglycone group and sugar. Steroidal saponins have various physiological activities, such as hypoglycemic, antiplatelet aggregation, anti-inflammatory, antitumor, antimicrobial, and enzyme activity inhibition, which is one of the key reasons why *Allium* has such significant health benefits. The structural diversity and rich biological activities of steroidal saponins make *Allium* important plants for both food and medicine. In this paper, the chemical structures, biological activities, and structure–activity relationships of steroidal saponins isolated from *Allium* are reviewed, and the biosynthetic pathways of some key compounds are proposed as well, to provide a molecular reference basis based on secondary metabolites for the health value of *Allium*.

## 1. Introduction

Supplementing nutrition from the diet is a guarantee for human bodies to maintain health. *Allium* plays an indispensable role in people’s diets, whether it is directly eaten as a vegetable or pickled condiments. The unique taste of natural *Allium* enhances people’s appetite, and its abundant biological activities bring people health and nutrition. *A. sativum* (garlic), for example, is a nutritious vegetable that is widely used as a condiment throughout the world. Fresh garlic bulbs contain about 65% water, 28% carbohydrates, 2.3% organic sulfur compounds, 2% protein, and 1.2% free amino acids (e.g., arginine, glycine, and cystine). Garlic contains 146 kcal/100 g of edible parts. It is also a rich source of vitamin C. There are 10–78.8 mg of this vitamin in 100 g of the edible parts of the product. Garlic also contains minerals—relatively high amounts of potassium, iron, and phosphorus (373–1367 mg, 1.5–13 mg, and 88–522 mg, respectively, in 100 g of the product) [1]. *Allium* plants usually have medicinal value, which can prevent and treat diseases. *A. sativum* is believed to have antibacterial, antioxidant, hypotension, and other effects and is used to treat influenza and hypertension.

*Allium* is a widespread perennial herbaceous plant and is one of the largest monocotyledonous genera. There are various species of *Allium*. However, the classification of *Allium* is still uncertain. Scholars generally believe that *Allium* belongs to Liliaceae in the broad sense at present. The classic Chinese works *Flora Reipublicae Popularis Sinicae*, *Flora of China*, and *Higher Plants of China* all follow Engler’s view of the plant classification system to deal with the systematic position of *Allium* and have assigned it to the broad family Liliaceae. The Angiosperm Phylogeny Group (APG) system of plant classification is a modern system of plant taxonomy. In APG III, the genus *Allium* belongs to the family Alliaceae under the family Amaryllidaceae, with more than 500 species, which is the largest genus in the family.

*Allium* plants are mostly distributed in temperate climates of the Northern Hemisphere, except for a few species occurring in Chile (e.g., *A. juncifolium*), Brazil (e.g., *A. sellovianum*), and tropical Africa (e.g., *A. spathaceum* and *A. dregeanum*). *Allium* plants are perennial bulb plants, with a few species such as *A. fistulosum* and *A. ampeloprasum* developing thickened leaf bases rather than forming bulbs. *Allium* plants are important cash crops, often with edible or medicinal value. For example, leeks (*A. tuberosum*), garlic (*A. sativum*), scallions (*A. fistulosum*), and onions (*A. cepa*) are common seasoning vegetables, while leek seeds and roots are used for medicinal purposes. Some species are also used as ornamental flowers, such as *A. cristophii* and *A. giganteum*. *Allium* plants usually have a peculiarly irritating odor due to the organosulfur compounds they produce. The main secondary metabolites of *Allium* plants are steroidal saponins. In addition, they also produce polysaccharides, proteins, phenolics, and other components. Steroidal saponins have been shown to have a variety of important biological effects and are thought to be one of the key reasons why *Allium* has such significant health benefits.

Steroidal saponins are a class of oligoglycosides, with spirostanes as the basic skeleton bonded to sugars, converted through the MVA or MEP pathway, and they are widely distributed in monocotyledonous plants, such as Liliaceae, Dioscoreaceae, and Agave, and less in dicotyledonous plants. As steroidal sapogenins are the raw materials for the synthesis of steroidal contraceptives and hormonal drugs, scholars have been studying steroidal saponin components in depth and have gradually found that most of them have good biological activities, such as hypoglycemic, antithrombotic, anti-inflammatory, antitumor, antimicrobial, and immune function-enhancing effects. By incorporating *Allium* into our diet, we can potentially reduce the risk of chronic diseases and improve our overall health.

A large number of steroidal saponins with favorable biological activities were isolated from *Allium*, but systematic and comprehensive comparison and biological activity mechanism studies are still lacking. A summary of the chemical structure of these compounds is necessary because it provides important information about their functional groups, stereochemistry, and sugar-linking sequence, which is essential for understanding their biological activities and potential therapeutic functions. At the same time, understanding their structure helps to identify and isolate these compounds from natural plant sources, as well as synthesize analogs for structure–activity relationship studies, thereby identifying key structural features of their biological activities and designing more effective and selective compounds. In addition, a summary of the biosynthetic pathways of these compounds will provide insight into the key enzymes and intermediates involved in the biosynthesis of these compounds, which will help optimize the production of steroidal saponins for medical and industrial applications. Therefore, this paper reviews the chemical compounds, biological activities, and structure–activity relationships of steroidal saponins from *Allium* reported in the literature and proposes the biosynthetic pathways of some key compounds to further explore the health value and therapeutic function of *Allium* vegetables from the molecular level of secondary metabolites.

## 2. Chemical Structures of *Allium* Steroidal Saponins

Steroidal saponins of *Allium* can be classified into three major categories according to the existence of E and F rings: furostane (F ring cleavage), spirostane (E/F rings into spiral rings), and cholestane (with C-17 side chains rather than oxygenated spiral rings) saponins, in addition to a few special glycoside types of steroidal saponins. The A/B ring of steroidal saponins is *cis* or *trans* (5-*β* and 5-*α*), the B/C ring is *trans*, the C/D ring is *trans*, and the D/E ring is *cis*. The C-10 and C-13 positions are connected to *β*-CH_3_, the C-20 is connected to *α*-CH_3_, and the C-25 position has two configurations of *R* and *S*. Steroidal saponins are commonly attached to straight chain sugar chains or branched sugar chains at C-3, and the types of saccharides are commonly glucose, galactose, rhamnose, xylose, and arabinose. The number of saccharides attached to steroidal saponins from *Allium* varies from one to five. All steroidal saponins isolated from *Allium* reported in the literature are shown in Table 1.

### 2.1. Furostane Saponins/Sapogenins

**Figure 1:**
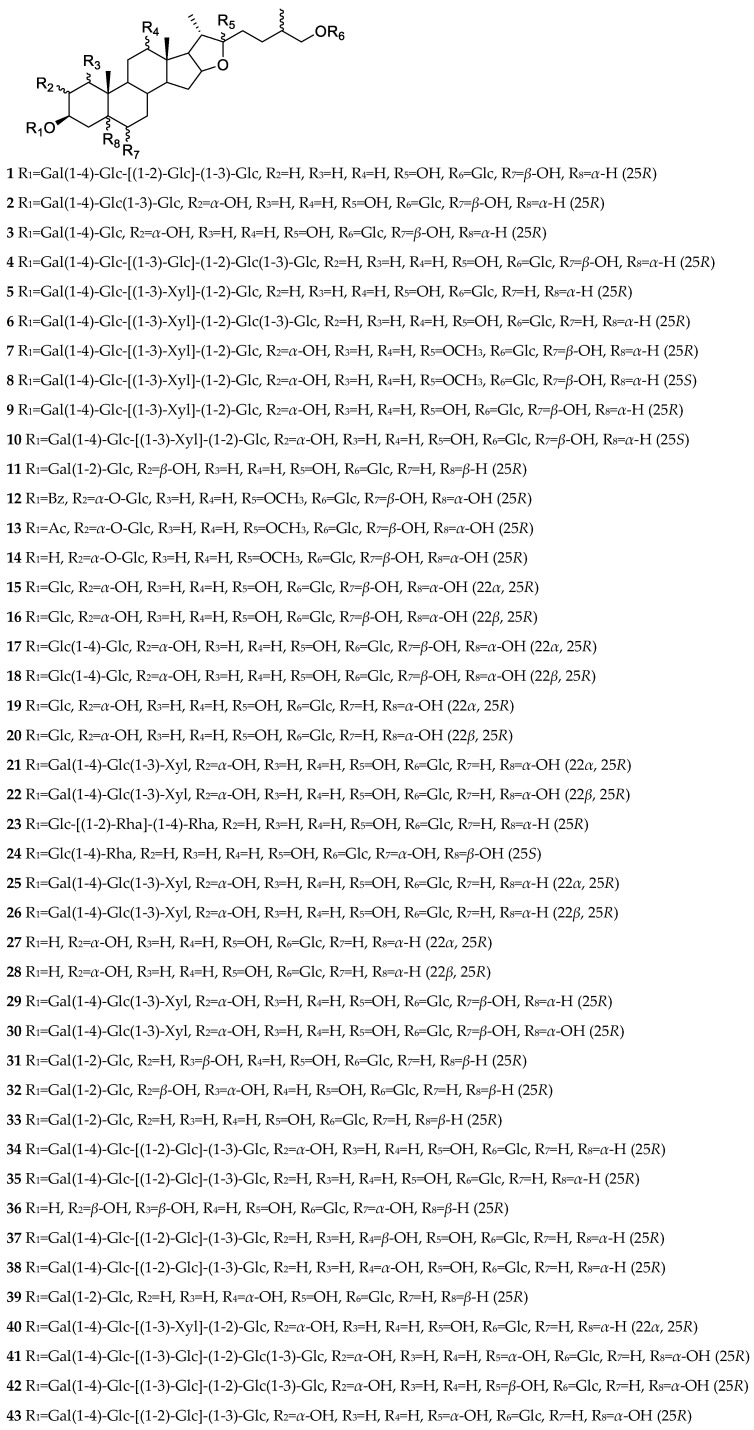

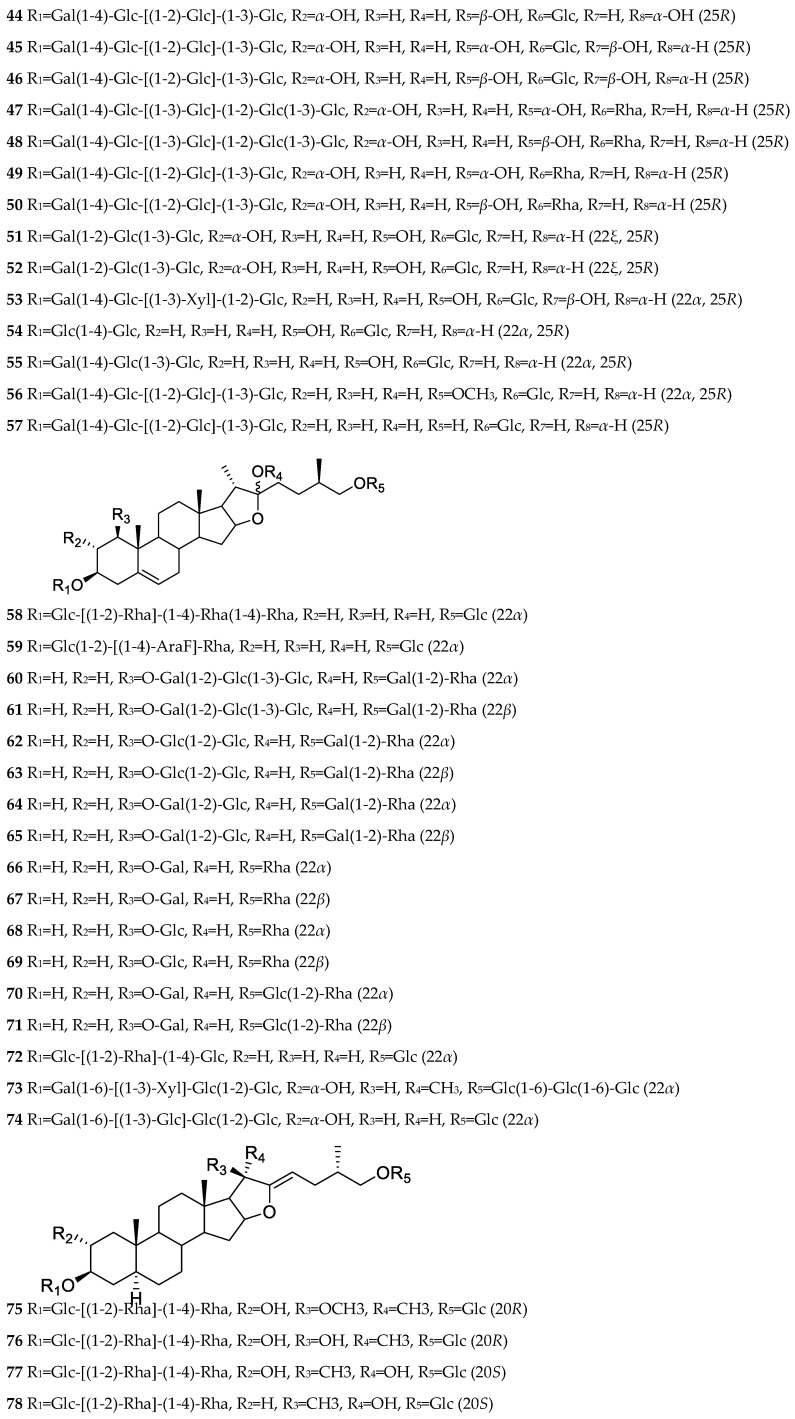

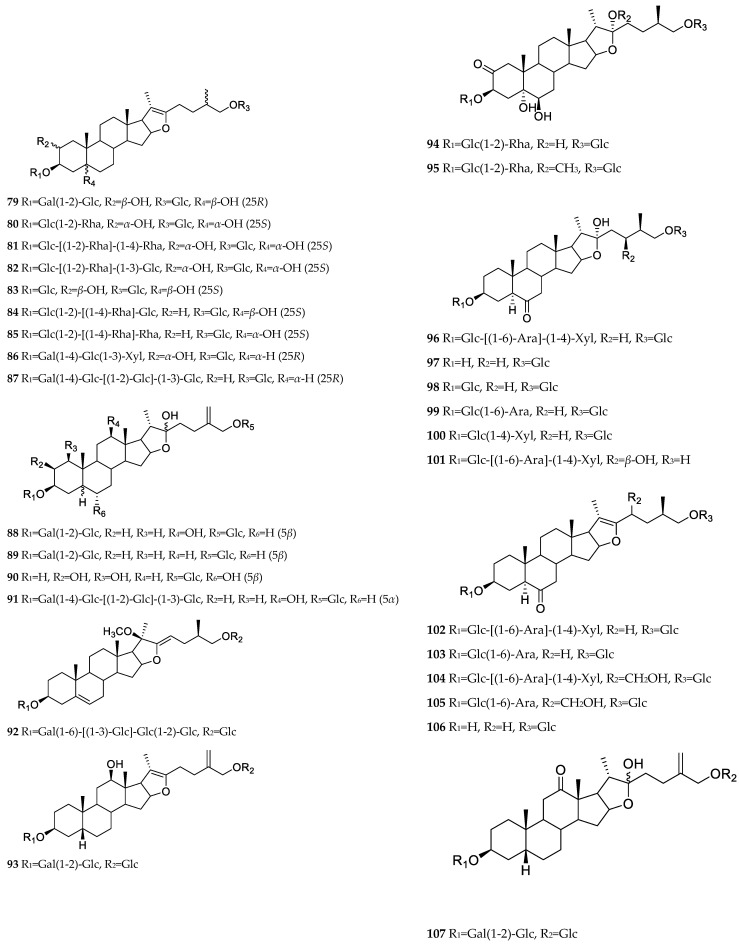
Furostane saponins/sapogenins isolated from *Allium* plants in recent years.

Furostane saponins usually have a saturated sapogenin, and its F ring is split. Some furostane saponins form double bonds at C-5(6), C-20(22), C-22(23), and C-25(27), or carbonyl groups at C-2, C-6, and C-12. The C-22 position of furostane saponins has *α* and *β* configurations. The sugar chains are often attached to C-3, C-26, C-2, and C-1. C-26 is mostly attached to monosaccharides and a few to disaccharides, in particular, C-26 of compound **73** isolated from the bulbs of *A. karataviense* is linked to trisaccharide [25]. The chemical structures of furostane saponins isolated from *Allium* in recent years are shown in Figure 1.

### 2.2. Spirostane Saponins/Sapogenins

**Figure 2:**
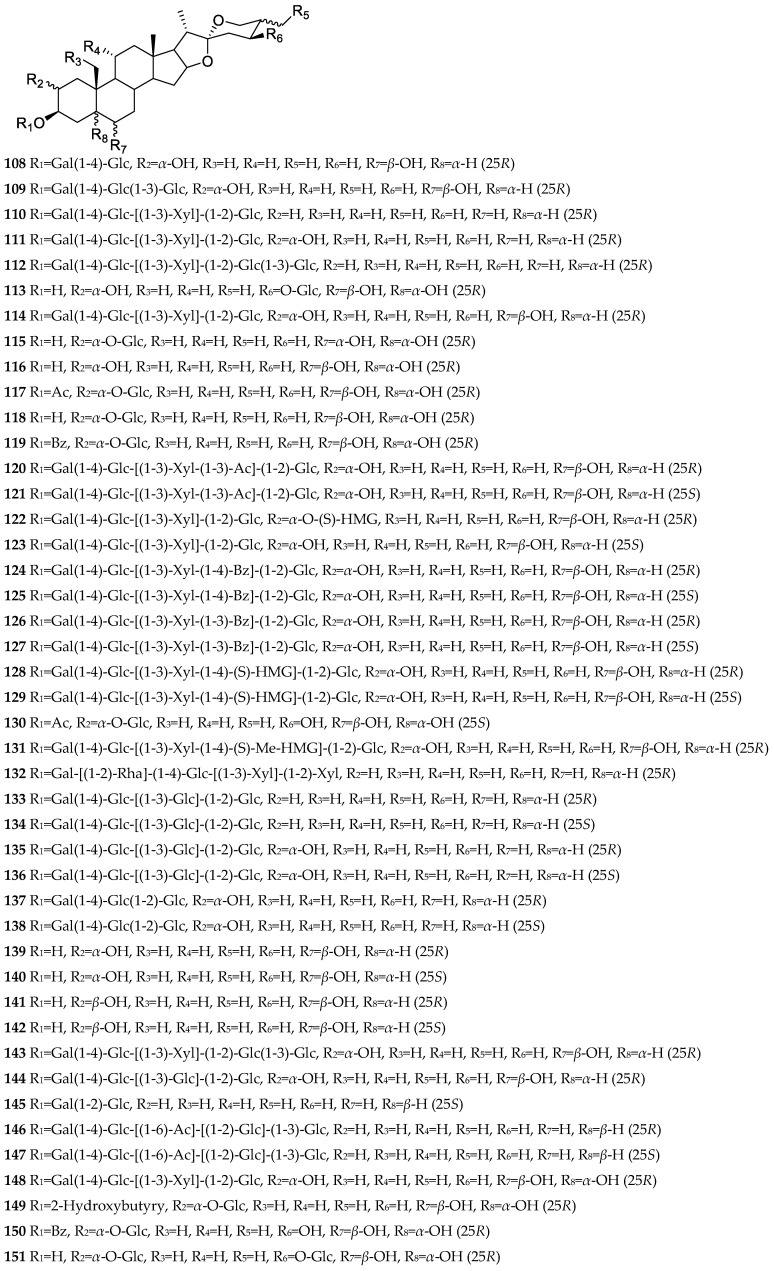

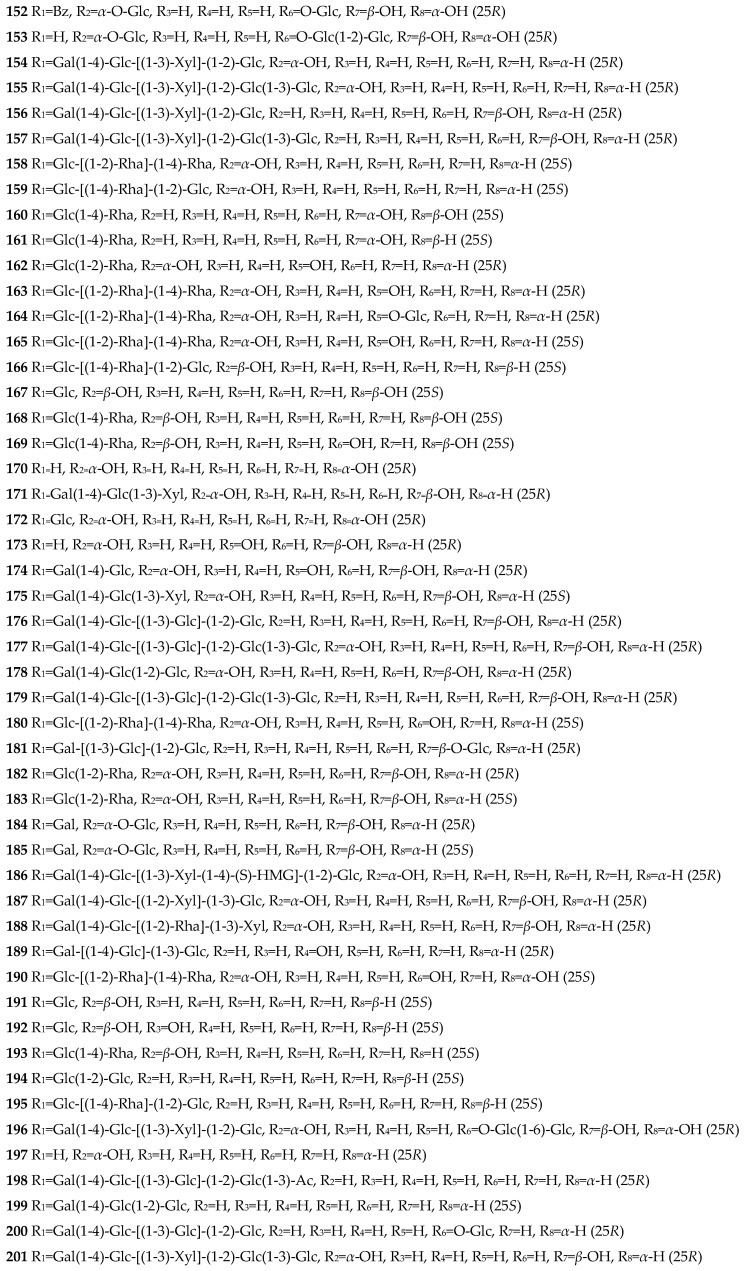

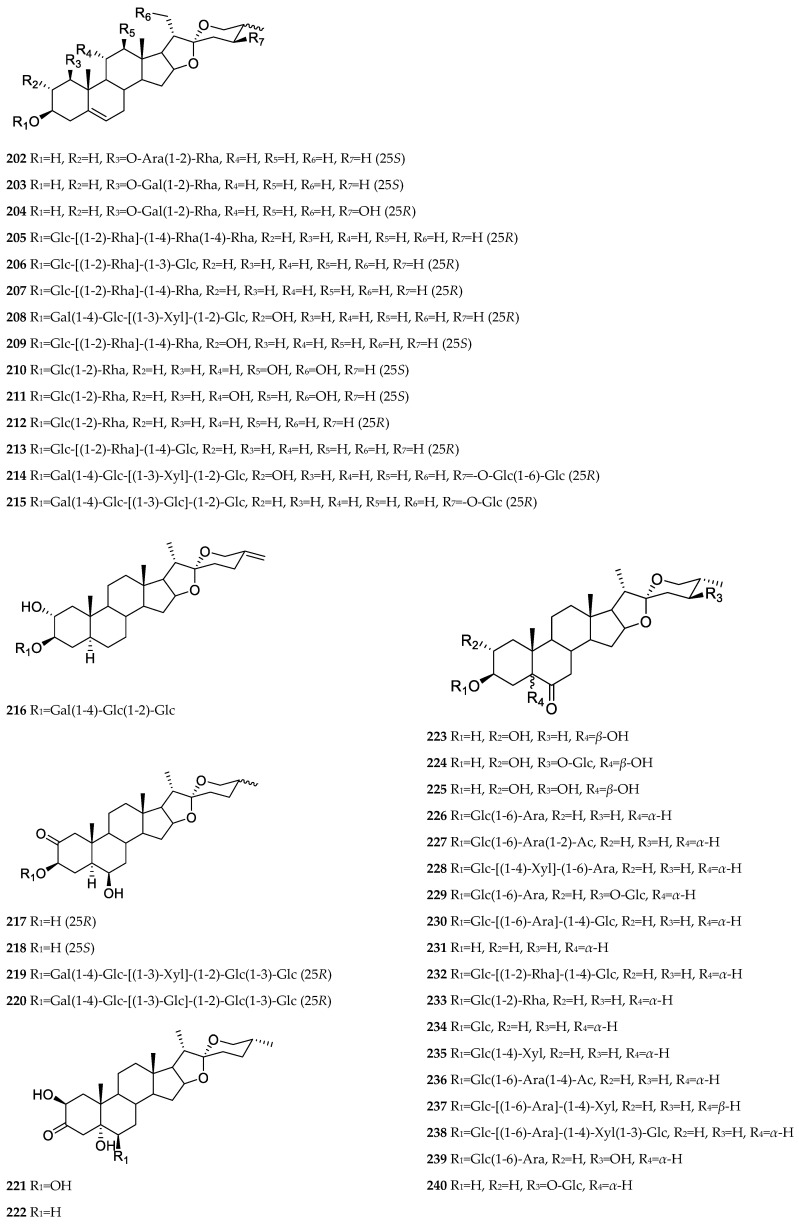

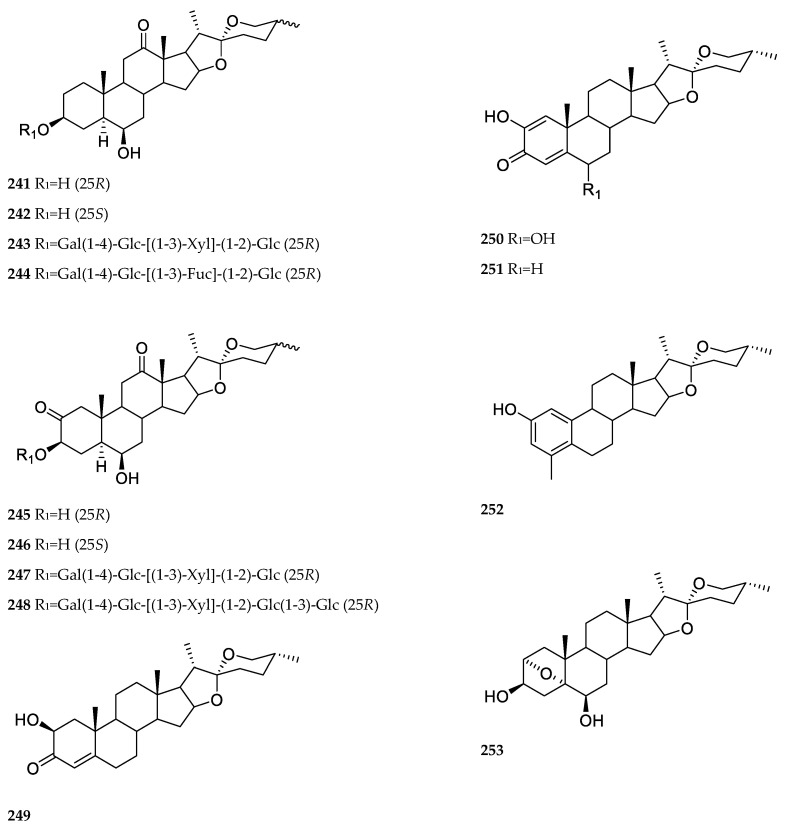
Spirostane saponins/sapogenins isolated from *Allium* plants in recent years.

Most spirostane saponins have a saturated spirostane skeleton, except that some saponins form carbonyl groups at C-2, C-3, C-6, C-12, or double bonds at C-5(6) and C-25(27). Notably, the A ring of compound **252** is aromatized, compound **249** forms a cyclohexenone structure at the A ring, compounds **250** and **251** form a cyclohexadienone structure at the A ring [73], and compound **253** forms an oxygen bridge between C-2 and C-5 [74]. The sugar moiety is commonly linked to C-2, C-3, and C-24, while compound **164** forms a glycosidic bond at C-27 [53], and compound **181** forms a glycosidic bond at C-6 [59]. The chemical structures of spirostane saponins isolated from *Allium* in recent years are shown in Figure 2.

### 2.3. Cholestane Saponins/Sapogenins

**Figure 3:**
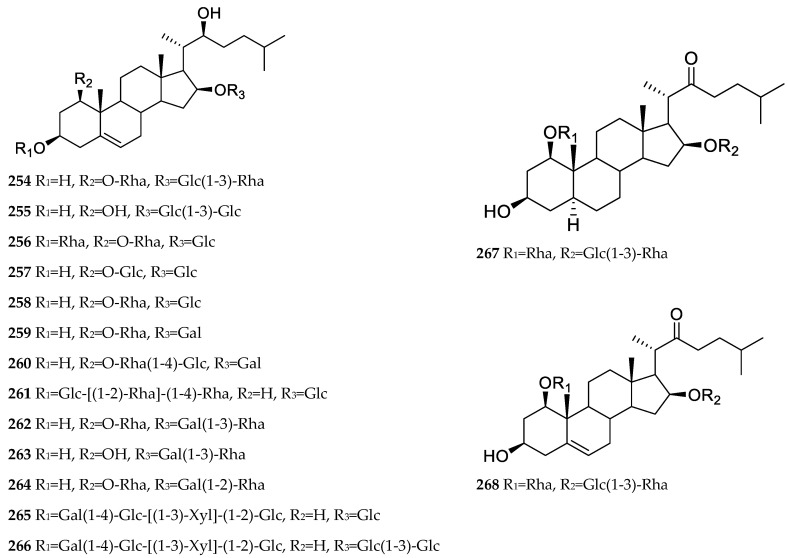
Cholestane saponins isolated from *Allium* plants in recent years.

Cholestane saponins, also known as open-chain saponins, usually have double bonds at C-5(6) and are oxidized at C-1, C-3, C-16, and C-22, except that compound **267** has no double bond at C-5(6) [5], and the C-1 of compounds **261**, **265**, and **266** are not oxidized [25,29]. This class of compounds usually forms glycosidic bonds at C-1, C-3, and C-16. The chemical structures of cholestane saponins isolated from *Allium* in recent years are shown in Figure 3.

### 2.4. Other Types of Steroidal Saponins/Sapogenins

**Figure 4:**
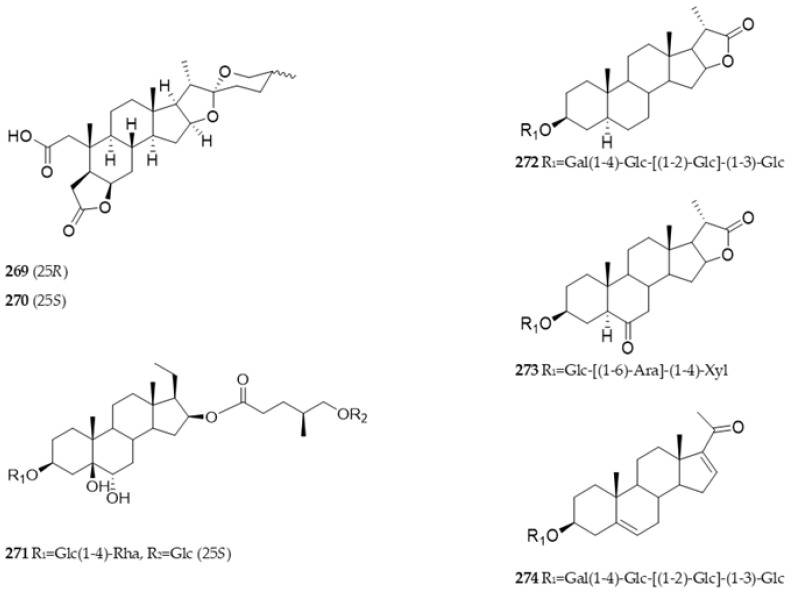
Other types of saponins/sapogenins isolated from *Allium* plants in recent years.

In addition to the three main types mentioned above, there are some steroidal saponins with rare skeletons. Compounds **271** and **274** are derived from pregnane [11,26]. Compounds **269** and **270** have an open A-ring and form a lactone ring at C-5 and C-6 [69]. Compounds **272** and **273** form a lactone ring structure at the E ring [13]. The chemical structures of other types of saponins isolated from *Allium* in recent years are shown in Figure 4.

## 3. Biological Activities of *Allium* Steroidal Saponins

Now it is generally believed that *Allium* exerts good biological activities due to the presence of organosulfur compounds and steroidal saponins. However, sulfur-containing compounds are much less stable than steroidal saponins, so it is of certain significance to study the biological activities of steroidal saponins of *Allium*. Modern pharmacological studies have demonstrated the hypoglycemic, antiplatelet aggregation, anti-inflammatory and other activities of these components through in vitro and in vivo experiments, confirming the medicinal value of *Allium*.

### 3.1. Hypoglycemic Effect

Visfatin is an insulin-mimetic adipocytokine that acts synergistically with insulin to enhance glucose uptake in vivo and in vitro and to inhibit the breakdown of liver glycogen into glucose, with insulin resistance-reducing and antidiabetic effects. Compound **133** isolated from *A. macrostemon* Bunge can increase the transcription of visfatin partly through the p38 MAPK pathway in differentiated 3T3-L1 adipocytes, which in turn increases the mRNA expression of visfatin and enhances the synthesis and secretion of the visfatin protein, thus having a hypoglycemic effect [47].

### 3.2. Antiplatelet Aggregation Effect

Adenosine diphosphate (ADP) can cause platelet aggregation through ADP receptors on platelet membranes, leading to thrombosis. Thrombus often causes angina pectoris, myocardial infarction, cerebral infarction, pulmonary embolism, and other acute diseases. *A. macrostemon* Bunge is a common traditional Chinese medicine used to treat cardiovascular diseases in China, as well as a common edible vegetable, and experiments have shown that the compound **88** isolated from this plant can inhibit platelet aggregation induced by ADP in vitro with an IC_50_ of 0.871 mM, confirming the plausibility of the pharmacological effect of this plant [30].

### 3.3. Gastroprotective Effect

Compounds **177** and **181** from the bulbs of *A. ampeloprasum* var. *porrum*, a Brazilian vegetable, have been proven to have anti-gastric ulcerative effects [57,59]. They may show protective properties on gastric cells by interfering with the ulcerogenesis mechanism, protecting the gastric mucosa, and reducing gastric congestion caused by acidified ethanol-induced acute gastric injury.

### 3.4. Immune Adjuvant Effect

Immune adjuvants are non-specific immune-enhancing substances that are injected into the body in advance or simultaneously with antigens and enhance the response of the body to the antigen or change the type of response. The immune adjuvant effect of compound **220**, which was isolated from *A. ampeloprasum* L. var. *porrum*, was higher than that of commercial adjuvants Freund’s Complete Adjuvant (FCA) and Freund’s Incomplete Adjuvant (FIA) in mice with ovalbumin (OVA) as an antigen by the delayed-type hypersensitivity (DTH) method [64]. The immune adjuvant effect of this compound may be due to its amphiphilic structure with hydrophilic sugar chains and lipophilic glycosides, which form adjuvant–antigen complexes and enhance antigen delivery to antigen-presenting cells for processing.

### 3.5. Anti-Inflammatory Effect

Ten compounds from the bulbs of *A. chinense* were evaluated for their anti-inflammatory effects by inhibiting NO production induced by lipopolysaccharide (LPS) in RAW 264.7 cells, and it was found that the NO inhibitory activity of steroidal saponins was related to both aglycones and sugar chains [16]. Compounds **34** and **54** showed significant anti-inflammatory effects with IC_50_ values of 2.01 ± 1.40 μM and 2.49 ± 1.54 μM, respectively. The compounds **225** and **230**, also from the bulbs of *A. chinense* showed anti-inflammatory activity with IC_50_ values of 32.20 ± 0.65 μM and 34.33 ± 5.04 μM [61]. Comparison with other compounds isolated in the same period showed that the A/B ring was active in *trans* and inactive in *cis*. The carbonyl group at C-6 increased the activity, while the hydroxylation at C-24 made the activity disappear, and the sugar chain at C-3 also affected the anti-inflammatory activity.

The anti-inflammatory effects of compounds **177** and **181** from the bulbs of *A. ampeloprasum* var. *porrum* were evaluated using an in vivo model of acute inflammation formed by foot swelling caused by carrageenan gum, and both compounds showed significant anti-inflammatory effects by rapidly controlling both stages of inflammation [57,59]. PCR was used to detect the inhibitory effects of compounds **37**, **38**, and **39** from the bulbs of *A. macrostemon* Bunge on the expression of CD40 ligand (CD40L) on the surface of platelet membranes activated by ADP stimulation [18]. Compounds **37** and **38** were able to significantly inhibit CD40L expression in a dose-dependent manner, indicating that they could be used as CD40L inhibitors for the treatment of platelet inflammation.

**Figure 5:**
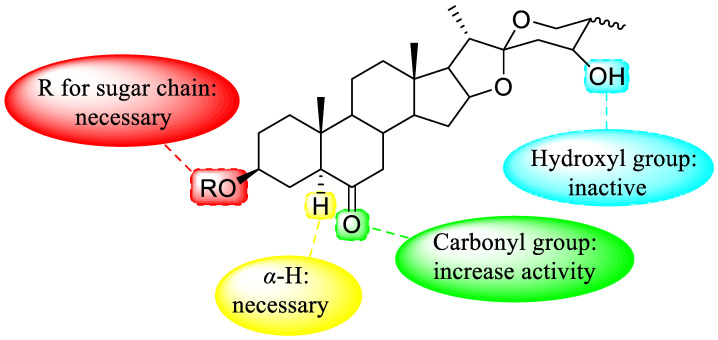
Effect of structure–activity relationship of steroidal saponins on anti-inflammatory effect.

Figure 5 presents the effect of the structure–activity relationship of steroidal saponins on the anti-inflammatory effect.

### 3.6. Cytotoxicity and Antitumor Effects

Studies have shown that some steroidal saponins from *Allium* are cytotoxic to tumor cells and may serve as drug candidates to treat cancer. Currently, it has been reported that steroidal saponins have significant antitumor activity on more than twenty types of tumor cells, such as A549 (human lung cancer cells), AGS (human gastric gland cancer cells), CNE-1 (human nasopharyngeal cancer cells), DLD-1 (human colorectal cancer cells), HeLa (human cervical cancer cells), HepG2 (human liver cancer cells), HT-29 (human colon cancer cells), MCF-7 (human breast cancer cells), etc.

The cytotoxicity of steroidal saponins is influenced by both aglycone and sugar chains. Compounds **111**, **155**, and **247** of spirostane saponins and compounds **259** and **260** of cholestane saponins from the bulbs of *A. porrum* L. exhibited strong cytotoxicity against both J-774 (IC_50_ 3.7, 2.1, 5.8, 4.6, and 4.0 μg/mL, respectively) and WEHI-164 cells (IC_50_ 4.8, 1.9, 4.3, 5.8, and 5.4 μg/mL, respectively), although a lack of activity of cholestane saponin was previously reported in the literature [36]. Compounds **111**, **114,** and **201** from the flowers of *A. porrum* L. were cytotoxic to mouse peritoneal cells, with an IC_50_ ranging from 5.70 to 11.13 μM [37]. Compounds **133**, **134, 137**, **138**, **198**, **199**, and **216** from the bulbs of *A. chinense* exhibited significant cytotoxicity against HepG2, A549, SPC-A-1 (human lung adenocarcinoma cells), CNE-1, and MGC80-3 (human gastric adenocarcinoma cells), with an IC_50_ ranging from 1.43 to 22.32 μM [61]. Compound **199** was not cytotoxic to MRC-5 (human embryonic lung fibroblasts), and its antitumor effects were selective. Comparison with other compounds isolated at the same time reveals that the configuration of H at C-5 and the selective acetylation of the hydroxyl group of sugar significantly affected the cytotoxicity. Beyond that, the polar oxygen-containing group (C=O) at C-6 of the B ring and the hydroxyl group at C-24 of the F ring led to a decrease in antitumor activity. Compounds **34**, **54**, **55**, and **56**, also from the bulbs of *A. chinense*, showed significant inhibitory effects on HepG2,2 A549, SPC-A-1, MGC80-3, MDA-MB-231(human breast cancer cells), SW620 (human colon cancer cell), and CNE-1, with IC_50_ values ranging from 1.76 to 22.83 μM [16]. Compound **54** also exhibited selective inhibitory effects and showed no cytotoxicity to MRC-5, and it could exert antitumor effects by inducing HepG2 cell cycle arrest and apoptosis. The relationship between steroidal saponins’ cytotoxicity on normal cells and the pharmacological activities, such as anti-inflammation and immune enhancement, deserves more in-depth study.

**Figure 6:**
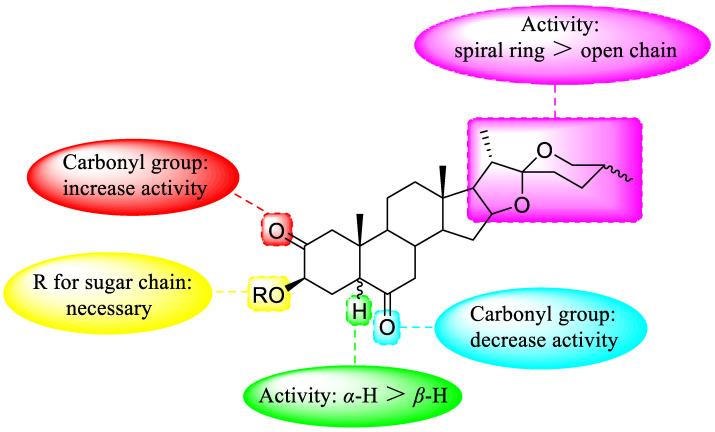
Effect of structure–activity relationship of steroidal saponins on cytotoxicity.

Figure 6 presents the effect of the structure–activity relationship of steroidal saponins on cytotoxicity.

### 3.7. Antimicrobial Effect

Steroidal saponins usually have the effect of inhibiting fungi and bacteria. Several studies have investigated the antifungal effect of steroidal saponins isolated from *Allium*. The reported fungal species include *Aspergillus niger*, *Alternaria alternata*, *Alternaria porri*, *Botrytis cinerea*, *Candida albicans, Fusarium solani*, *Fusarium oxysporum*, *Fusarium oxysporum* f. sp. *lycopersici*, *Mortierella ramanniana*, *Penicillium italicum*, *Pythium ultimum*, *Rhizoctonia solani*, *Trichoderma harzianum*, *Trichoderma harzianum* (strains P1), and *Trichoderma harzianum* (strain T39). Compared to fungi, bacteria are usually less sensitive to saponin components, and fewer species of bacteria have been reported to be inhibited by steroidal saponins from *Allium*, including *Bacillus subtilis*, *Escherichia coli*, etc.

Compounds **29**, **30**, **116**, **140**, and **175** from the bulbs of *Allium minutiflorum* Regel inhibited ten soil-borne fungal pathogens, including *Fusarium oxysporum*, *Fusarium oxysporum* f. sp. *lycopersici*, *Fusarium solani*, and biocontrol fungi, but not against bacteria such as *Xanthomonas campestris* pv. *Campestris*, *Agrobacterium tumefaciens*, and *Streptomyces turgidiscabies* [14]. Compound **175,** with a high content in bulbs (83.5 mg/kg), showed the strongest antifungal activity. A structure–activity relationship analysis revealed that the spirostane skeleton was more active than the furostane skeleton, and the hydroxyl group at C-5 of the furostane saponins promoted antifungal activity. Compounds **187** and **188** from the seeds of *Allium ampeloprasum* subsp. *Persicum* showed antifungal activity against *Penicillium italicum*, *Aspergillus niger*, and *Trichoderma harzianum*, while other steroidal saponins isolated at the same time showed no antifungal activity, indicating that the spirostane skeleton was more active than the furostane and cholestane skeletons [21]. This also confirms that the hydroxyl group at C-6 could enhance the antifungal activity.

Six spirostane saponins from the roots of *Allium tuberosum* were evaluated for their antibacterial effects against *Bacillus subtilis* and *Escherichia coli*, and the results showed that compound **192** exhibited good antibacterial activity (MIC 64 μg/mL, both), compounds **194** (MIC 16 and 32 μg/mL, respectively) and **195** (MIC 16 μg/mL, both) showed potent antibacterial activity, while compounds **167**, **191**, and **193** showed almost no bacterial inhibitory effect (MIC > 128 μg/mL) [55]. A structure–activity relationship analysis revealed that the sugar at C-3 and the hydroxyl group at C-19 enhanced the antibacterial activity, while the hydroxyl groups at C-2 and C-5 attenuated the antibacterial activity.

**Figure 7:**
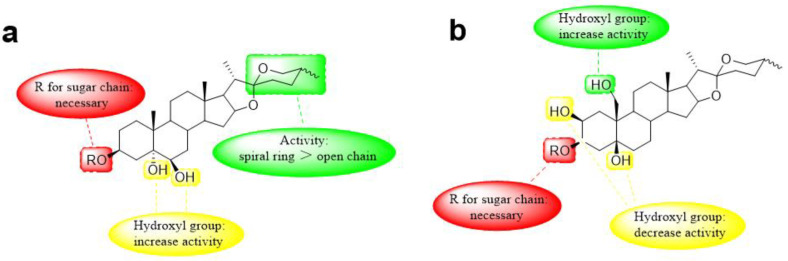
Effect of structure–activity relationship of steroidal saponins on antimicrobial effect. (**a**) Effect of structure–activity relationship on antifungal activity. (**b**) Effect of structure–activity relationship on antibacterial activity.

Figure 7 presents the effects of steroidal saponins on antifungal and antibacterial activities, respectively.

### 3.8. Enzyme Activity Inhibition Effect

Na,K-ATPase is responsible for the active transport of Na^+^ and K^+^ in cells, and drugs that inhibit Na,K-ATPase activity can be used to treat diseases associated with ion active transport disorders for which the transporter enzyme is responsible. Fourteen compounds from the collective fruit of *A. karataviense* Rgl and *A. cepa* L were tested for their inhibition of Na,K-ATPase activity [62]. Compounds **202**, **203**, and **204** had the strongest enzyme inhibitory activity, with IC_50_ values of 1.0 × 10^−5^ M, 3.4 × 10^−5^ M, and 8.8 × 10^−5^ M. Furthermore, compounds **202** and **203** had non-competitive inhibition, and compound **204** had competitive inhibition. The results of the structure–activity analysis show that the hydroxyl group at C-24 of the F ring decreased the inhibitory activity, the carbonyl group at C-6 of the steroid skeleton slightly enhanced the inhibitory activity, and when the F ring was opened and the sugar was connected to the hydroxyl group at C-25, the inhibitory activity was weakened, and even enzyme activity was promoted. In addition, compounds **133** and **134** from the bulbs of *A. chinense* also inhibited Na, K-ATPase, with an IC_50_ of 4 × 10^−5^ M [46].

cAMP phosphodiesterases are enzymes that catabolize cyclic adenosine acids under the activation of calmodulin bound to Ca^2+^. The inhibition of cAMP phosphodiesterase increases intracellular cAMP content, which enhances myocardial contraction, dilates peripheral blood vessels, and improves heart failure. Compounds **133**, **134**, **226**, **227**, and **228** from the bulbs of *A. chinense* significantly reduced the activity of cAMP phosphodiesterase, especially compound **227**, with an IC_50_ of 3.3 × 10^−5^ M, which is comparable to the positive drug papaverine (IC_50_ 3.0 × 10^−5^ M) [46].

The compounds **7**, **12**, **13**, **114**, **117**, **128**, and **130** from the bulbs of *A. giganteum* can significantly inhibit the activity of cAMP phosphodiesterase [6]. A structure–activity relationship analysis revealed that the inhibition activity was enhanced when the (S)-3-hydroxy-3-methylglutaroyl (HMG group) was attached to the hydroxyl group at C-4 of xylose. Furostane steroidal saponins **7**, **12**, and **13** exhibited stronger activity than the corresponding spirostane steroidal saponins **114** and **117**, which may be related to the multiple hydroxyl groups in their A and B rings.

### 3.9. Antispasmodic Effect

Compounds **27**, **28**, and **172** isolated from the flowers of *A. hirtifolium* Boiss and compounds **15**, **16**, **19**, **20**, and **170** isolated from the bulbs of *A. elburzense* were tested for their antispasmodic activity by using the histamine-induced contractions of isolated ileum of guinea-pig as a model [12]. Compounds **19**, **20**, and **170** exhibited the strongest inhibitory contractile activity. A structure–activity relationship analysis showed that the antispasmodic activity can be enhanced by the hydroxyl group at C-5 and glucose at C-26, while it can be attenuated by the hydroxyl group at C-6 and glucose at C-3.

### 3.10. Cardiomyocyte Regulation Effect

Calcium ions play an important role in the regulation of cell physiological function. Under normal physiological conditions, the intracellular calcium levels are relatively stable and in dynamic regulation; otherwise, it may lead to cell damage or apoptosis. Compounds **33**, **35**, **36**, and **90** isolated from *A. macrostemon* further increased KCl-induced [Ca^2+^]_i_ increase in guinea pig cardiomyocytes, suggesting that it may enhance myocardial function [17]. In contrast, compound **87** inhibited the KCl-induced [Ca^2+^]_i_ increase, suggesting its potential for the treatment of heart failure.

### 3.11. Nerve Cell Protection Effect

The antioxidant activity was investigated using the CCK-8 method for six compounds isolated from *A. chinense* G. Don, and hydrogen peroxide was selected to induce a neuronal oxidative damage model in PC12 cells [13]. Compared to five other compounds isolated simultaneously, compound **57** had a significant protective effect on neuronal cell injury induced by hydrogen peroxide in a dose-dependent manner, which may be related to its H at C-22 and the higher amounts of glucose and galactose in the sugar chain.

### 3.12. Hemolysis Effect

Steroidal saponins can bind to cholesterol on the erythrocyte membrane to form insoluble complexes, disrupting the osmotic pressure of the cell membrane and inducing hemolysis. Usually, spirostane saponins have stronger hemolytic activity due to a higher affinity with cholesterol. Compounds **177**, **181**, and **220** isolated from the bulbs of *A. ampeloprasum* var. *porrum*, have all shown hemolytic ability, with HD_50_ values of 20 μg/mL, 6.5 μg/mL, and 13 μg/mL, respectively [57,59,64]. Their hemolysis is on the same order of magnitude as that of the reference commercial saponin QS-21 isolated from *Quillaja saponaria* (HD_50_ 5 μg/mL).

## 4. Proposed Biosynthetic Pathways of *Allium* Steroidal Saponins

The biosynthetic pathway of steroid saponins is acetyl coenzyme A through the mevalonate (MVA) pathway or pyruvate through the methylerythritol 4-phosphate (MEP) pathway to produce farnesyl pyrophosphate (FPP), which is then catalyzed by squalene synthase (SQS) to produce squalene, and by squalene epoxidase (SQE) to produce 2, 3-oxidized squalene, and then catalyzed by cycloatenol synthase (CAS) to produce cycloatenol, which is the precursor of steroid compounds. The cycloatenol further synthesizes cholestanol, which is then hydroxylated at C-22, C-26, and C-16 to form semi-ketal structure compounds, which in turn synthesizes furostane saponin. The glycosidic bond at C-26 of furostane saponin is easily hydrolyzed, and after hydrolysis, it cyclizes into a spiral ketal structure to synthesize spirostane saponin.

**Figure 8:**
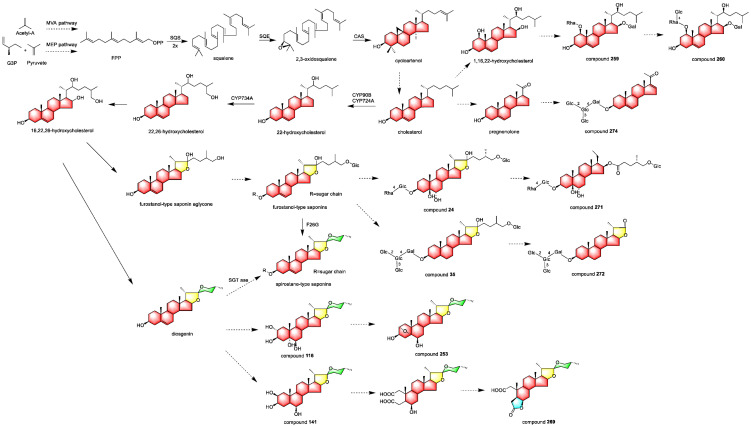
Proposed biosynthetic pathways of *Allium* steroidal saponins (MVA: mevalonate; MEP: methylerythritol 4-phosphate).

Cholestane compounds **259** and **260** can be produced by the glycosylation of 1,16,22-trihydroxycholestrol. Pregnane compound **274** can be synthesized from cholesterol by multi-step oxidative breakage to produce pregnenolone, which in turn synthesizes compound **274** by glycosylation. Compound **271** can be synthesized from furostane compound **24** by oxidative breakage at C-22, and compound **272** can be synthesized from furostane compound **35** by a similar mechanism. The oxygen bridge of the A-ring of spirostane compound **253** can be synthesized by the dehydration cyclization of compound **116**. Compound **269** can be synthesized from compound **141** by oxidation at C-2 and C-3, and then C-3 is dehydrated with C-6 to form a lactone ring. The proposed biosynthetic pathways of *Allium* steroidal saponin are shown in Figure 8.

## 5. Conclusions

In recent decades, as common functional vegetables, a wide variety of *Allium* plants have been extensively studied for their important edible and medicinal values. Undoubtedly, their secondary metabolite, a steroidal saponin, is one of the most important chemical bases for the healthcare functions of *Allium*. In this paper, we have summarized the chemical constituents, biological activities, and structure–activity relationships of steroidal saponins isolated from *Allium* and have proposed the biosynthetic pathways of some key compounds to clarify the molecular basis of the rich health function of *Allium* as a vegetable and condiment from the perspective of secondary metabolites. In addition, we have explored the positive role of *Allium* in the prevention and treatment of diseases more comprehensively.

## Figures and Tables

**Table 1:** Steroidal saponins/sapogenins isolated from *Allium* plants reported in the literature.

No.	Common Name	Structure Name	Species	Parts	References
1	proto-eruboside-B	26-O-*β*-glucopyranosyl 22-hydroxy-25(*R*)-5*α*-furostane-3*β*,6*β*-26-triol 3-O-*β*-glucopyranosyl-(1→2)-[*β*-glucopyranosyl-(1→3)]-*β*-glucopyranosyl-(1→4)-*β*-galactopyranoside	*A. sativum* L	bulbs	[2]
2	ampeloside Bf1	(25*R*)-26-O-*β*-glucopyranosyl-22-hydroxy-5*α*-furostane-2*α*,3*β*,6*β*,26-tetraol-3-O-*β*-glucopyranosyl-(1→3)-*β*-glucopyranosyl-(1→4)-*β*-galactopyranoside	*A. ampeloprasum* L	bulbs	[3]
3	ampeloside Bf2	(25*R*)-26-O-*β*-glucopyranosyl-22-hydroxy-5*α*-furostane-2*α*,3*β*,6*β*,26-tetraol-3-O-*β*-glucopyranosyl-(1→4)-*β*-galactopyranoside	*A. ampeloprasum* L	bulbs	[3]
4	sativoside-B1	(25*R*)-26-O-*β*-D-glucopyranosyl-22-hydroxy-5*α*-furostane-3*β*,6*β*,26-triol 3-O-*β*-D-glucopyranosyl-(1→3)-O-*β*-D-glucopyranosyl-(1→2)-O-[*β*-D-glucopyranosyl-(1→3)]-O-*β*-D-glucopyranosyl-(1→4)-O-*β*-D-galactopyranoside	*A. sativum* L	bulbs	[4]
5	proto-desgalactotigonin		*A. sativum* L	bulbs and roots	[4]
6	sativoside-R1	(25*R*)-26-O-*β*-D-glucopyranosyl-22-hydroxy-5*α*-furostane-3*β*,26-diol 3-O-*β*-D-glucopyranosyl-(1→3)-O-*β*-D-glucopyranosyl-(1→2)-O-[*β*-D-xylopyranosyl-(1→3)]-O-*β*-D-glucopyranosyl-(1→4)-O-*β*-D-galactopyranosyde	*A. sativum* L	roots	[4]
7		22-O-methyl-26-O-*β*-D-glucopyranosyl-(25*R*)-5*α*-furostane-2*α*,3*β*,6*β*,22*ξ*,26-pentol 3-O-{O-*β*-D-glucopyranosyl-(1→2)-O-[*β*-D-xylopyranosyl-(1→3)]-O-*β*-D-glucopyranosyl-(1→4)-*β*-D-galactopyranoside}	*A. albopilosum*	bulbs	[5]
			*A. ostrowskianum*	bulbs	[5]
			*A. giganteum*	bulbs	[6]
8		26-O-*β*-D-glucopyranosyl-(25*S*)-5*α*-furostane-2*α*,3*β*,6*β*,22*ξ*,26-pentaol 3-O-{O-*β*-D-glucopyranosyl-(1→2)-O-[*β*-D-xylopyranosyl-(1→3)]-O-*β*-D-glucopyranosyl-(1→4)-*β*-D-galactopyranoside}	*A. albopilosum*	bulbs	[5]
			*A. ostrowskianum*	bulbs	[5]
9		26-O-*β*-D-glucopyranosyl-(25*R*)-5*α*-furostan-2*α*,3*β*,6*β*,22*ξ*,26-pentol 3-O-*β*-D-glucopyranosyl-(1→2)-O-[*β*-D-xylopyranosyl-(1→3)]-O-*β*-D-glucopyranosyl-(1→4)-*β*-D-galactopyranoside	*A. schubertii*	bulbs	[7]
10		26-O-*β*-D-glucopyranosyl-(25*S*)-5*α*-furostan-2*α*,3*β*,6*β*,22*ξ*,26-pentol 3-O-*β*-D-glucopyranosyl-(l→2)-O-[*β*-D-xylopyranosyl-(1→3)]-O-*β*-D-glucopyranosyl-(1→4)-*β*-D-galactopyranoside	*A. schubertii*	bulbs	[7]
11	macrostemonoside J	26-O-*β*-D-glucopyranosyl 2*β*,3*β*,22,26-tetrahydroxy-25(*R*)-5*β*-furostan 3-O-*β*-D-glucopyranosyl (1→2)-*β*-D-galactopyranoside	*A. macrostemon* Bunge	bulbs	[8]
12		3-O-benzoyl-22-O-methyl-26-O-*β*-D-glucopyranosyl-(25*R*)-5*α*-furostane-2*α*,3*β*,5*α*,6*β*,22*ξ*,26-hexol 2-O-*β*-D-glucopyranoside	*A. giganteum*	bulbs	[6]
13		3-O-acetyl-22-O-methyl-26-O-*β*-D-glucopyranosyl-(25*R*)-5*α*-furostane-2*α*,3*β*,5*α*,6*β*,22*ξ*,26-hexol 2-O-*β*-D-glucopyranoside	*A. giganteum*	bulbs	[6]
14		(25*R*)-26-O-*β*-D-glucopyranosyl-22-O-methyl-5*α*-furostane-2*α*,3*β*,5,6*β*,22*ξ*-pentol 2-O-*β*-D-glucopyranoside	*A. karataviense*	bulbs	[9]
15	elburzensosides A1	furost-2*α*,3*β*,5*α*,6*β*,22*α*-pentol 3-O-*β*-D-glucopyranosyl 26-O-*β*-D-glucopyranoside	*A. elburzense*	bulbs	[10]
16	elburzensosides A2	furost-2*α*,3*β*,5*α*,6*β*,22*β*-pentol 3-O-*β*-D-glucopyranosyl 26-O-*β*-D-glucopyranoside	*A. elburzense*	bulbs	[10]
17	elburzensosides B1	furost-2*α*,3*β*,5*α*,6*β*,22*α*-pentol 3-O-[*β*-D-glucopyranosyl-(1→4)-O-*β*-D-glucopyranosyl] 26-O-*β*-D-glucopyranoside	*A. elburzense*	bulbs	[10]
18	elburzensosides B2	furost-2*α*,3*β*,5*α*,6*β*,22*β*-pentol 3-O-[*β*-D-glucopyranosyl-(1→4)-O-*β*-D-glucopyranosyl] 26-O-*β*-D-glucopyranoside	*A. elburzense*	bulbs	[10]
19	elburzensosides C1	furost-2*α*,3*β*,5*α*,22*α*-tetrol 3-O-*β*-D-glucopyranosyl 26-O-*β*-D-glucopyranoside	*A. elburzense*	bulbs	[10]
20	elburzensosides C2	furost-2*α*,3*β*,5*α*,22*β*-tetrol 3-O-*β*-D-glucopyranosyl 26-O-*β*-D-glucopyranoside	*A. elburzense*	bulbs	[10]
21	elburzensosides D1	furost-2*α*,3*β*,5*α*,22*α*-tetrol 3-O-[*β*-D-xylopyranosyl-(1→3)-O-*β*-D-glucopyranosyl-(1→4)-O-*β*-D-galactopyranosyl] 26-O-*β*-D-glucopyranoside	*A. elburzense*	bulbs	[10]
22	elburzensosides D2	furost-2*α*,3*β*,5*α*,22*β*-tetrol 3-O-[*β*-D-xylopyranosyl-(1→3)-O-*β*-D-glucopyranosyl-(1→4)-O-*β*-D-galactopyranosyl] 26-O-*β*-D-glucopyranoside	*A. elburzense*	bulbs	[10]
23		26-O-*β*-D-glucopyranosyl-(25*R*)-3*β*,22*ξ*,26-trihydroxyl-5*α*-furostane 3-O-*β*-chacotrioside	*A. tuberosum* Rottler	seeds	[11]
24		26-O-*β*-D-glucopyranosyl-(25*S*)-3*β*,5*β*,6*α*,22*ξ*,26-pentahydroxyl-5*β*-furostane 3-O-*α*-L-rhamnopyranosyl-(1→4)-*β*-D-glucopyranoside	*A. tuberosum* Rottler	seeds	[11]
25	hirtifolioside A1	furost-2*α*,3*β*,22*α*-triol 3-O-[*β*-D-xylopyranosyl-(1→3)-O-*β*-D-glucopyranosyl-(1→4)-O-*β*-D-galactopyranosyl]-26-O-*β*-D-glucopyranoside	*A. hirtifolium* Boiss	flowers	[12]
26	hirtifolioside A2	furost-2*α*,3*β*,22*β*-triol 3-O-[*β*-D-xylopyranosyl-(1→3)-O-*β*-D-glucopyranosyl-(1→4)-O-*β*-D-galactopyranosyl]-26-O-*β*-D-glucopyranoside	*A. hirtifolium* Boiss	flowers	[12]
27	hirtifolioside C1	(25*R*)-5*α*-furostane-2*α*,3*β*,22*α*,26-tetraol-26-O-*β*-D-glucopyranoside	*A. hirtifolium* Boiss	flowers	[12]
			*A. chinense* G. Don	bulbs	[13]
28	hirtifolioside C2	furost-2*α*,3*β*,22*β*-triol 26-O-*β*-D-glucopyranoside	*A. hirtifolium* Boiss	flowers	[12]
29	minutoside A	(25*R*)-furost-2*α*,3*β*,6*β*,22*α*,26-pentaol 3-O-[*β*-D-xylopyranosyl-(1→3)-O-*β*-D-glucopyranosyl-(1→4)-O-*β*-D-galactopyranosyl] 26-O-*β*-D-glucopyranoside	*A. minutiflorum* Regel	bulbs	[14]
30	minutoside C	(25*R*)-furost-2*α*,3*β*,5*α*,6*β*,22*α*,26-esaol 3-O-[*β*-D-xylopyranosyl-(1→3)-O-*β*-D-glucopyranosyl-(1→4)-O-*β*-D-galactopyranosyl] 26-O-*β*-D-glucopyranoside	*A. minutiflorum* Regel	bulbs	[14]
31	macrostemonoside P	(25*R*)-26-O-*β*-D-glucopyranosyl-22-hydroxy-5*β*-furostane-1*β*,3*β*, 26-triol-3-O-*β*-D-glucopyranosyl (1→2)-*β*-D-galactopyranoside	*A. macrostemon* Bunge	bulbs	[15]
32	macrostemonoside Q	(25*R*)-26-O-*β*-D-glucopyranosyl-22-hydroxy-5*β*-furost-1*α*,2*β*,3*β*, 26-tetraol-3-O-*β*-D-glucopyranosyl (1→2)-*β*-D-galactopyranoside	*A. macrostemon* Bunge	bulbs	[15]
33		(25*R*)-26-O-*β*-D-glucopyranosyl-22-hydroxy-5*β*-furostane-3*β*, 26-diol-3-O-*β*-D-glucopyranosyl (1→2)-*β*-D-galactopyranoside	*A. macrostemon* Bunge	bulbs	[15]
34	macrostemonoside R	(25*R*)-26-O-*β*-D-glucopyranosyl-22-hydroxy-furostane-2*α*,3*β*,26-triol-3-O-*β*-D-glucopyranosyl (1→2)-[*β*-D-glucopyranosyl (1→3)]-*β*-D-glucopyranosyl (1→4)-*β*-D-galactopyranoside	*A. macrostemon* Bunge	bulbs	[15]
			*A. chinense* G. Don	bulbs	[16]
35	macrostemonoside B		*A. macrostemon* Bunge	bulbs	[15]
36	macrostemonoside M	(25*R*)-22-hydroxy-5*β*-furostane-1*β*,2*β*,3*β*,6*α*-tetraol-26-O-*β*-D-glucopyranoside	*A. macrostemon* Bunge	bulbs	[17]
37		(25*R*)-26-O-*β*-D-glucopyranosyl-5*α*-furostane-3*β*,12*β*,22,26-tetraol-3-O-*β*-D-glucopyranosyl (1→2) [*β*-D-glucopyranosyl (1 →3)]-*β*-D-glucopyranosyl (1 →4)-*β*-D-galactopyranoside	*A. macrostemon* Bunge	bulbs	[18]
38		(25*R*)-26-O-*β*-D-glucopyranosyl-5*α*-furostane-3*β*,12*α*,22,26-tetraol-3-O-*β*-D-glucopyranosyl (1→2) [*β*-D-glucopyranosyl (1→3)]-*β*-D-glucopyranosyl (1 →4) -*β*-D-galactopyranoside	*A. macrostemon* Bunge	bulbs	[18]
39		(25*R*)-26-O-*β*-D-glucopyranosyl-5*β*-furostane-3*β*,12*α*,22,26-tetraol-3-O-*β*-D-glucopyranosyl (1→2)-*β*-D-galactopyranoside	*A. macrostemon* Bunge	bulbs	[18]
40		26-O-*β*-D-glucopyranosyl-(25*R*)-5*α*-furostan-2*α*,3*β*,22*α*,26-tetraol 3-O-*β*-D-glucopyranosyl-(1→2)[*β*-D-xylopyranosyl-(1→3)]-*β*-D-glucopyranosyl-(1→4)-*β*-D-galactopyranoside	*A. rotundum*	inflorescences and flower stalks	[19]
41	voghieroside A1	furosta-2*α*,3*β*,5*α*,22*α*,26-pentol 3-O-*β*-D-glucopyranosyl-(1→3)-*β*-D-glucopyranosyl-(1→2)-[*β*-D-glucopyranosyl-(1→3)]-*β*-D-glucopyranosyl-(1→4)-*β*-D-galactopyranosyl-26-O-*β*-D-glucopyranoside	*A. sativum* L. var. Voghiera	bulbs	[20]
42	voghieroside A2	furosta-2*α*,3*β*,5*α*,22*β*,26-pentol 3-O-*β*-D-glucopyranosyl-(1→3)-*β*-D-glucopyranosyl-(1→2)-[*β*-D-glucopyranosyl-(1→3)]-*β*-D-glucopyranosyl-(1→4)-*β*-D-galactopyranosyl-26-O-*β*-D-glucopyranoside	*A. sativum* L. var. Voghiera	bulbs	[20]
43	voghieroside B1	furosta-2*α*,3*β*,5*α*,22*α*,26-pentol 3-O-*β*-D-glucopyranosyl-(1→2)-[*β*-D-glucopyranosyl-(1→3)]-*β*-D-glucopyranosyl-(1→4)-*β*-D-galactopyranosyl-26-O-*β*-D-glucopyranoside	*A. sativum* L. var. Voghiera	bulbs	[20]
44	voghieroside B2	furosta-2*α*,3*β*,5*α*,22*β*,26-pentol 3-O-*β*-D-glucopyranosyl-(1→2)-[*β*-D-glucopyranosyl-(1→3)]-*β*-D-glucopyranosyl-(1→4)-*β*-D-galactopyranosyl-26-O-*β*-D-glucopyranoside	*A. sativum* L. var. Voghiera	bulbs	[20]
45	voghieroside C1	furosta-2*α*,3*β*,6*β*,22*α*,26-pentol 3-O-*β*-D-glucopyranosyl-(1→2)-[*β*-D-glucopyranosyl-(1→3)]-*β*-D-glucopyranosyl-(1→4)-*β*-D-galactopyranosyl -26-O-*β*-D-glucopyranoside	*A. sativum* L. var. Voghiera	bulbs	[20]
46	voghieroside C2	furosta-2*α*,3*β*,6*β*,22*β*,26-pentol 3-O-*β*-D-glucopyranosyl-(1→2)-[*β*-D-glucopyranosyl-(1→3)]-*β*-D-glucopyranosyl-(1→4)-*β*-D-galactopyranosyl -26-O-*β*-D-glucopyranoside	*A. sativum* L. var. Voghiera	bulbs	[20]
47	voghieroside D1	furosta-2*α*,3*β*,22*α*,26-tetrol 3-O-*β*-D-glucopyranosyl-(1→3)-*β*-D-glucopyranosyl-(1→2)-[*β*-D-glucopyranosyl-(1→3)]-*β*-D-glucopyranosyl-(1→4)-*β*-D-galactopyranosyl-26-O-*α*-L-rhamnopyranoside	*A. sativum* L. var. Voghiera	bulbs	[20]
48	voghieroside D2	furosta-2*α*,3*β*,22*β*,26-tetrol 3-O-*β*-D-glucopyranosyl-(1→3)-*β*-D-glucopyranosyl-(1→2)-[*β*-D-glucopyranosyl-(1→3)]-*β*-D-glucopyranosyl-(1→4)-*β*-D-galactopyranosyl-26-O-*α*-L-rhamnopyranoside	*A. sativum* L. var. Voghiera	bulbs	[20]
49	voghieroside E1	furosta-2*α*,3*β*,22*α*,26-tetrol 3-O-*β*-D-glucopyranosyl-(1→2)-[*β*-D-glucopyranosyl-(1→3)]-*β*-D-glucopyranosyl-(1→4)-*β*-D-galactopyranosyl-26-O-*α*-L-rhamnopyranoside	*A. sativum* L. var. Voghiera	bulbs	[20]
50	voghieroside E2	furosta-2*α*,3*β*,22*β*,26-tetrol 3-O-*β*-D-glucopyranosyl-(1→2)-[*β*-D-glucopyranosyl-(1→3)]-*β*-D-glucopyranosyl-(1→4)-*β*-D-galactopyranosyl-26-O-*α*-L-rhamnopyranoside	*A. sativum* L. var. Voghiera	bulbs	[20]
51	persicoside D1	furosta-2*α*,3*β*,22*ξ*,26-tetraol 3-O-*β*-D-glucopyranosyl (1→3)-*β*-D-glucopyranosyl (1→2)-*β*-D-galactopyranosyl 26-O-*β*-D-glucopyranoside	*A. ampeloprasum* subsp. *persicum*	seeds	[21]
52	persicoside D2	furosta-2*α*,3*β*,22*ξ*,26-tetraol 3-O-*β*-D-glucopyranosyl (1→3)-*β*-D-glucopyranosyl (1→2)-*β*-D-galactopyranosyl 26-O-*β*-D-glucopyranoside	*A. ampeloprasum* subsp. *persicum*	seeds	[21]
53	leucofuranoside A	26-O-*β*-D-glucopyranosyl-(25*R*)-5*α*-furostane-3*β*,6*β*-diol-3-O-*β*-D-glucopyranosyl-(1→2)-O-*β*-D-xylopyranosyl-(1→3)-O-*β*-D-glucopyranosyl-(1→4)-*β*-D-galactopyranoside	*A. leucanthum*	flowers	[22]
54		(25*R*)-26-O-*β*-D-glucopyranosyl-5*α*-furost-3-*β*,26-didyroxy-3-O-{O-*β*-D-glucopyranosyl-(1→4)-*β*-D-galactopyranoside}	*A. chinense* G. Don	bulbs	[16]
55	tomatoside A		*A. chinense* G. Don	bulbs	[16]
56	macorstemonoside C		*A. chinense* G. Don	bulbs	[16]
57		(25*R*)-26-O- *β* -D-glucopyranosyl-5*α* -furostane-3*β*,26-diol-3-O-*β*-D-glucopyranosyl-(1→2)-[*β*-D-glucopyranosyl-(1→3)]-*β*-D-glucopyranosyl-(1→4)-*β*-D-galacopyranoside	*A. chinense* G. Don	bulbs	[13]
58	dichotomin	(25*R*)-26-O-*β*-D-glucopyranosyl-22-hydroxy-5-ene-furostan-3*β*, 26-diol-3-O-*α*-L-rhamnopyranosyl-(1→4)-*α*-L-rhamnopyranosyl-(1→4)-[*α*-L-rhamnopyranosyl-(1→2)]-*β*-D-glucopyranoside	*A. ascalonicum* L		[23]
59	parisaponin	(25*R*)-26-O-*β*-D-glucopyranosyl-22-hydroxy-5-ene-furostan-3*β*, 26-diol-3-O-*α*-L-rhamnopyranosyl-(1→2)-[*α*-L-arabinofuranosyl-(1→4)]-*β*-D-glucopyranoside	*A. ascalonicum* L		[23]
60	persicoside C1	furosta-1*β*,3*β*,22*ξ*,26-tetraol 5-en 1-O-*β*-D-glucopyranosyl (1→3)-*β*-D-glucopyranosyl (1→2)-*β*-D-galactopyranosyl 26-O-*α*-L-rhamnopyranosyl (1→2)-*β*-D-galactopyranoside	*A. ampeloprasum* subsp. *persicum*	seeds	[21]
61	persicoside C2	furosta-1*β*,3*β*,22*ξ*,26-tetraol 5-en 1-O-*β*-D-glucopyranosyl (1→3)-*β*-D-glucopyranosyl (1→2)-*β*-D-galactopyranosyl 26-O-*α*-L-rhamnopyranosyl (1→2)-*β*-D-galactopyranoside	*A. ampeloprasum* subsp. *persicum*	seeds	[21]
62	ceposide A1		*A. ampeloprasum* subsp. *persicum*	seeds	[21]
63	ceposide A2		*A. ampeloprasum* subsp. *persicum*	seeds	[21]
64	ceposide C1		*A. ampeloprasum* subsp. *persicum*	seeds	[21]
65	ceposide C2		*A. ampeloprasum* subsp. *persicum*	seeds	[21]
66	tropeoside A1		*A. ampeloprasum* subsp. *persicum*	seeds	[21]
67	tropeoside A2		*A. ampeloprasum* subsp. *persicum*	seeds	[21]
68	tropeoside B1		*A. ampeloprasum* subsp. *persicum*	seeds	[21]
69	tropeoside B2		*A. ampeloprasum* subsp. *persicum*	seeds	[21]
70	ascalonicoside A1		*A. ampeloprasum* subsp. *persicum*	seeds	[21]
71	ascalonicoside A2		*A. ampeloprasum* subsp. *persicum*	seeds	[21]
72	deltoside	(25*R*)-furost-5-en-3*β*,22*α*,26-triol 26-O-*β*-D-glucopyranosyl-3-O-*α*-L-rhamnopyranosyl-(1→2)-[*β*-D-glucopyranosyl-(1→4)]-*β*-D-glucopyranoside	*A. schoenoprasum*	whole plants	[24]
73	karatavioside G	(25*R*)-26-[(O-*β*-D-glucopyranosyl-(1→6)-*β*-D-glucopyranosyl-(1→6)-*β*-D-glucopyranosyl)oxy]-2*α*-hydroxy-22*α*-methoxyfurost-5-en-3*β*-yl O-*β*-D-glucopyranosyl-(1→2)-[*β*-D-xylopyranosyl-(1→3)]*β*-D-glucopyranosyl-(1→4)-*β*-D-galactopyranoside	*A. karataviense*	bulbs	[25]
74	allimacroside D	(25*R*)-26-O-*β*-D-glucopyranosyl-5-enefurostan-2*α*,3*β*,22*α*,26-tetraol-3-O-*β*-D-glucopyranosyl(1→2)-[*β*-D-glucopyranosyl(1→3)]-*β*-D-glucopyranosyl(1→4)-*β*-D-galactopyranoside	*A. macrostemon* Bunge	whole plants	[26]
75	tuberoside F	26-O-*β*-D-glucopyranosyl-(25*S*,20*R*)-20-O-methyl-5*α*-furost-22(23)-en-2*α*,3*β*,20,26-tetraol 3-O-*α*-L-rhamnopyranosyl-(1→2)-[*α*-L-rhamnopyranosyl-(1→4)]-*β*-D-glucopyranoside	*A. tuberosum*	seeds	[27]
76	tuberoside G	26-O-*β*-D-glucopyranosyl-(25*S*,20*R*)-5*α*-furost-22(23)-en-2*α*,3*β*,20,26-tetraol 3-O-*α*-L-rhamnopyranosyl-(1→2)-[*α*-L- rhamnopyranosyl-(1→4)]-*β*-D-glucopyranoside	*A. tuberosum*	seeds	[27]
77	tuberoside H	26-O-*β*-D-glucopyranosyl-(25*S*,20*S*)-5*α*-furost-22(23)-en-2*α*,3*β*,20,26-tetraol 3-O-*α*-L-rhamnopyranosyl-(1→2)-[*α*-L-rhamnopyranosyl-(1→4)]-*β*-D-glucopyranoside	*A. tuberosum*	seeds	[27]
78	tuberoside I	26-O-*β*-D-glucopyranosyl-(25*S*,20*S*)-5*α*-furost-22(23)-en-3*β*,20,26-triol 3-O-*α*-L-rhamnopyranosyl-(1→2)-[*α*-L-rhamnopyranosyl-(1→4)]-*β*-D-glucopyranoside	*A. tuberosum*	seeds	[27]
79	macrostemonoside L	26-O-*β*-D-glucopyranosyl 2*β*,3*β*,26-trihydroxy-25(*R*)-5*β*-furostan-20(22)-ene 3-O-*β*-D-glucopyranosyl (1→2)-*β*-D-galactopyranoside	*A. macrostemon* Bunge	bulbs	[8]
80	tuberoside A	26-O-*β*-D-glucopyranosyl-(25*S*)-5*α*-furost-20(22)-ene-2*α*,3*β*,26-triol 3-O-*α*-L-rhamnopyranosyl-(1→2)-O-*β*-D-glucopyranoside	*A. tuberosum*	seeds	[28]
81	tuberoside B	26-O-*β*-D-glucopyranosyl-(25*S*)-5*α*-furost-20(22)-ene-2*α*,3*β*,26-triol 3-O-*α*-L-rhamnopyranosyl-(1→2)-[*α*-L-rhamnopyranosyl-(1→4)]-*β*-D-glucopyranoside	*A. tuberosum*	seeds	[28]
82	tuberoside C	26-O-*β*-D-glucopyranosyl-(25*S*)-5*α*-furost-20(22)-ene-2*α*,3*β*,26-triol 3-O-*α*-L-rhamnopyranosyl-(1→2)-[*β*-D-glucopyranosyl-(1→3)]-*β*-D-glucopyranoside	*A. tuberosum*	seeds	[28]
83	tuberoside R	26-O-*β*-D-glucopyranosyl-(25*S*)-5*β*-furost-20(22)-ene-2*β*,3*β*, 5, 26-tetraol 3-O-*β*-D-glucopyranoside	*A. tuberosum* L	seeds	[29]
84	tuberoside S	26-O-*β*-D-glucopyranosyl-(25*S*)-5*β*-furost-20(22)-ene-3*β*,26-diol 3-O-*β*-D-glucopyranosyl-(1→2)-[*α*-L-rhamnopyranosyl (1→4)]-*β*-D-glucopyranoside	*A. tuberosum* L	seeds	[29]
85	tuberoside T	26-O-*β*-D-glucopyranosyl-(25*S*)-5*α*-furost-20(22)-ene-3*β*, 26-diol 3-O-*α*-L-rhamnopyranosyl (1→2)-[*α*-L-rhamnopyranosyl (1→4)]-*β*-D-glucopyranoside	*A. tuberosum* L	seeds	[29]
86	hirtifolioside B	furost-20(22)-ene-2*α*,3*β*-diol 3-O-[*β*-D-xylopyranosyl-(1→3)-O-*β*-D-glucopyranosyl-(1→4)-O-*β*-D-galactopyranosyl]-26-O-*β*-D-glucopyranoside	*A. hirtifolium* Boiss	flowers	[12]
87	macrostemonoside E		*A. macrostemon* Bunge	bulbs	[17]
88	macrostemonoside G	26-O-*β*-D-glucopyranosyl-22-hydroxy-5*β*-furost-25(27)-ene-3*β*,12*β*,26-triol 3-O-*β*-D-glucopyranosyl(1→2)-*β*-D-galactopyranoside	*A. macrostemon* Bunge	bulbs	[30]
89	macrostemonoside O	26-O-*β*-D-glucopyranosyl-22-hydroxy-5-*β*-furost-25 (27)-ene-3*β*, 26-diol-3-O-*β*-D-glucopyranosyl (1→2)-*β*-D-galactopyranoside	*A. macrostemon* Bunge	bulbs	[15]
90	macrostemonoside N	22-hydroxy-5*β*-furost-25-(27)-ene-1*β*,2*β*,3*β*,6*α*-tetraol-26-O-*β*-D-glucopyranoside	*A. macrostemon* Bunge	bulbs	[17]
91		26-O-*β*-D-glucopyranosyl-5*α*-furost-25 (27)-ene-3*β*,12*β*,22,26-tetraol-3-O-*β*-D-glucopyranosyl-(1→2)-[*β*-D-glucopyranosyl-(1→3)]-*β*-D-glucopyranosyl-(1→4)-*β*-D-galactopyranoside	*A. macrostemon* Bunge	bulbs	[31]
92	allimacroside E	26-O-*β*-D-glucopyranosyl-20*β*-methoxyl-25(*R*)-furostan-5,22(23)-dien-3*β*,26-diol-3-O-*β*-D-glucopyranosyl(1→2)-[*β*-D-glucopyranosyl(1→3)]-*β*-D-glucopyranosyl(1→4)-*β*-D-galactopyranoside	*A. macrostemon* Bunge	whole plants	[26]
93		26-O-*β*-D-glucopyranosyl-5*β*-furost-20 (22)-25 (27)-dien-3*β*,12*β*,26-triol-3-O-*β*-D-glucopyranosyl-(1→2)-*β*-D-galactopyranoside	*A. macrostemon* Bunge	bulbs	[31]
94	ascalonicoside C	(25*R*)-26-O-*β*-D-glucopyranosyl-22-hydroxy-5*α*-furost-2-one-3*β*,5,6*β*, 26-tetraol-3-O-*α*-L-rhamnopyranosyl-(1→2)-*β*-D-glucopyranoside	*A. ascalonicum* L		[23]
95	ascalonicoside D	(25*R*)-26-O-*β*-D-glucopyranosyl-22-methoxy-5*α*-furost-2-one-3*β*,5,6*β*, 26-tetraol- 3-O-*α*-L-rhamnopyranosyl-(1→2)-*β*-D-glucopyranoside	*A. ascalonicum* L		[23]
96	chinenoside I	26-O-*β*-D-glucopyranosyl 3*β*,22,26-tridyroxy-25(*R*)-5*α*-furostan-6-one 3-O-*β*-D-xylopyranosyl(1→4)-[*α*-L-arabinopyranosyl (1→6)]-*β*-D-glucopyranoside	*A. chinense* G. Don	bulbs	[32]
97		26-O-*β*-D-glucopyranosyl 3*β*,22*α*,26-trihydroxy-25(*R*)-5*α*-furostan-6-one	*A. chinense* G. Don	bulbs	[16]
98		26-O-*β*-D-glucopyranosyl 3*β*,22*α*,26-trihydroxy-25(*R*)-5*α*-furostan-6-one 3-O-*β*-D-glucopyranoside	*A. chinense* G. Don	bulbs	[16]
99		26-O-*β*-D-glucopyranosyl 3*β*,22,26-tridyroxy 25(*R*)-5*α*-furostan-6-one 3-O-*α*-L-arabinopyranosyl(1→6)-*β*-D-glucopyranoside	*A. chinense* G. Don	bulbs	[16]
100		(25*R*)-6-ketone-26-O-*β*-D-glucopyranosyl-5*α*-furostane-3*β*,22*α*,26-triol-3-O-*α*-L-xylopyranosyl-(1→ 4)-*β*-D-glucopyranoside	*A. chinense* G. Don	bulbs	[13]
101		(25*R*)-6-ketone-5*α* -furostane-3*β*,22*α*,24*β*,26-tetraol-3-O-*β*-D-xylopyranosy-(1→4)-[*α*-L-arabinopyranosyl-(1→6)]-*β*-D-glucopyranoside	*A. chinense* G. Don	bulbs	[13]
102	chinenoside II	26-O-*β*-glucopyranosyl 3*β*,26-dihydroxy-(25*R*)-5*α*-furost-20(22)-en-6-one 3-O-*β*-xylopyranosyl-(1→4)-[*α*-arbinopyranosyl(1→6)]-*β*-glucopyranoside	*A. chinense* G. Don	bulbs	[33]
103	chinenoside III	26-O-*β*-glucopyranosyl 3*β*,26-dihydroxy-(25*R*)-5*α*-furost-20(22)-en-6-one 3-O-*α*-arabinopyranosyl(1→6)-*β*-glucopyranoside	*A. chinense* G. Don	bulbs	[33]
104	chinenoside IV	26-O-*β*-glucopyranosyl-3*β*,26-dihydroxy-23-hydroxymethyl-25(*R*)-5*α*-furost-20(22)-en-6-one 3-O-*β*-xylopyranosyl(1→4)-[*α*-arabinopyranosyl(1→6)]-*β*-glucopyranoside	*A. chinense* G. Don	bulbs	[34]
105	chinenoside V	26-O-*β*-glucopyranosyl-3*β*,26-dihydroxy-23-hydroxymethyl-25(*R*)-5*α*-furost-20(22)-en-6-one 3-O-*α*-arabinopyranosyl(1→6)-*β*-glucopyranoside	*A. chinense* G. Don	bulbs	[34]
106		26-O-*β*-D-glucopyranosyl 3*β*,26-dihydroxy-25(*R*)-5*α*-furostan-20(22)-en-6-one	*A. chinense* G. Don	bulbs	[16]
107	macrostemonoside I	26-O-*β*-D-glucopyranosyl-22-hydroxy-5*β*-furost-25(27)-ene12-one-3*β*,26-diol 3-O-*β*-D-glucopyranosyl(1→2)-*β*-D-galactopyranoside	*A. macrostemon* Bunge	bulbs	[30]
108		agigenin 3-O-*β*-glucopyranosyl-(1→4)-*β*-galactopyranoside	*A. ampeloprasum* L	bulbs	[3]
109	ampeloside Bs1	agigenin 3-O-*β*-glucopyranosyl-(1→3)-*β*-glucopyranosyl-(1→4)-*β*-galactopyranoside	*A. ampeloprasum* L	bulbs	[3]
			*A. sativum* L. var. Voghiera	bulbs	[20]
110	desgalactotigonin		*A. sativum* L	roots	[4]
111	F-gitonin	(25*R*)-5*α*-spirostane-2*α*,3*β*-diol 3-O-{O-*β*-D-glucopyranosyl-(1→2)-O-[*β*-D-xylopyranosyl-(1→3)]-O-*β*-D-glucopyranosyl-(1→4)-*β*-D-galactopyranoside}	*A. sativum* L	roots	[4]
			*A. ostrowskianum*	bulbs	[5]
			*A. jesdianum*	bulbs	[35]
			*A. porrum* L	bulbs	[36]
				flowers	[37]
			*A. cyrillii*	bulbs	[38]
112	sativoside-R2	tigogenin 3-O-*β*-D-glucopyranosyl-(1→3)-O-*β*-D-glucopyranosyl-(1→2)-O-[*β*-D-xylopyranosyl-(1→3)]-O-*β*-D-glucopyranosyl-(1→4)-O-*β*-D-galactopyranosyde	*A. sativum* L	roots	[4]
113		(24*S*,25*R*)-5*α*-spirostan-2*α*,3*β*,5*α*,6*β*,24-pentaol 24-O-*β*-D-glucopyranoside	*A. giganteum*	bulbs	[39]
114	aginoside	(25*R*)-5*α*-spirostan-2*α*,3*β*,6*β*-triol 3-O-*β*-D-glucopyranosyl-(1→2)-O-[*β*-D-xylopyranosyl-(1→3)]-O-*β*-D-glucopyranosyl-(1→4)-*β*-D-galactopyranoside	*A. giganteum*	bulbs	[39]
			*A. albopilosum*	bulbs	[5]
			*A. ostrowskianum*	bulbs	[5]
			*A. schubertii*	bulbs	[7]
			*A. macleanii*	bulbs	[40]
			*A. ampeloprasum* L	bulbs	[41]
			*A. jesdianum*	bulbs	[35]
			*A. leucanthum*	flowers	[42]
			*A. nigrum* L	bulbs	[43]
				root–bulb basal stem	[44]
			*A. porrum* L	flowers	[37]
115		(25*R*)-5*α*-spirostan-2*α*,3*β*,5*α*,6*α*-tetraol 2-O-*β*-D-glucopyranoside	*A. aflatunense*	bulbs	[39]
116	alliogenin	(25*R*)-5*α*-spirostan-2*α*,3*β*,5*α*,6*β*-tetraol	*A. giganteum*	bulbs	[45]
			*A. albopilosum*	bulbs	[5]
			*A. karataviense*	bulbs	[9]
			*A. minutiflorum* Regel	bulbs	[14]
117		(25*R*)-3-O-acetyl-5*α*-spirostan-2*α*,3*β*,5*α*,6*β*-tetraol 2-O-*β*-D-glucopyranoside	*A. giganteum*	bulbs	[45]
			*A. albopilosum*	bulbs	[5]
			*A. karataviense*	bulbs	[9]
118		(25*R*)-5*α*-spirostan-2*α*,3*β*,5*α*,6*β*-tetraol 2-O-*β*-D-glucopyranoside	*A. giganteum*	bulbs	[45]
			*A. albopilosum*	bulbs	[5]
			*A. macleanii*	bulbs	[40]
			*A. karataviense*	bulbs	[9]
119		(25*R*)-3-O-benzoyl-5*α*-spirostan-2*α*,3*β*,5*α*,6*β*-tetraol 2-O-*β*-D-glucopyranoside	*A. giganteum*	bulbs	[45]
			*A. macleanii*	bulbs	[40]
			*A. karataviense*	bulbs	[9]
120		(25*R*)-5*α*-spirostane-2*α*,3*β*,6*β*-triol 3-O-(O-*β*-D-glucopyranosyl-(1→2)-O-[3-O-acetyl-*β*-D-xylopyranosyl-(1→3)]-O-*β*-D-glucopyranosyl-(1→4)-*β*-D-galactopyranoside}	*A. albopilosum*	bulbs	[5]
121		(25*S*)-5*α*-spirostane-2*α*,3*β*,6*β*-triol 3-O-(O-*β*-D-glucopyranosyl-(1→2)-O-[3-O-acetyl-*β*-D-xylopyranosyl-(1→3)]-O-*β*-D-glucopyranosyl-(1→4)-*β*-D-galactopyranoside}	*A. albopilosum*	bulbs	[5]
122		(25*R*)-2-O-[(*S*)-3-hydroxy-3-methylglutaroyl]-5*α*-spirostane-2*α*,3*β*,6*β*-triol 3-O-{O-*β*-D-glucopyranosyl-(1→2)-O-[*β*-D-xylopyranosyl-(1→3)-O-*β*-D-glucopyranosyl-(1→4)-*β*-D-galactopyranoside}	*A. albopilosum*	bulbs	[5]
123	turoside A	(25*S*)-5*α*-spirostan-2*α*,3*β*,6*β*-triol 3-O-*β*-D-glucopyranosyl-(1→2)-O-[*β*-D-xylopyranosyl-(1→3)]-O-*β*-D-glucopyranosyl-(1→4)-*β*-D-galactopyranoside	*A. schubertii*	bulbs	[7]
			*A. nigrum* L	bulbs	[43]
				root–bulb basal stem	[44]
124		(25*R*)-5*α*-spirostan-2*α*,3*β*,6*β*-triol 3-O-*β*-D-glucopyranosyl-(1→2)-O-[4-O-benzoyl-*β*-D-xylopyranosyl-(1→3)]-O-*β*-D-glucopyranosyl-(1→4)-*β*-D-galactopyranoside	*A. schubertii*	bulbs	[7]
			*A. macleanii*	bulbs	[40]
125		(25*S*)-5*α*-spirostan-2*α*,3*β*,6*β*-triol 3-O-*β*-D-glucopyranosyl-(1→2)-O-[4-O-benzoyl-*β*-D-xylopyranosyl-(1→3)]-O-*β*-D-glucopyranosyl-(1→4)-*β*-D-galactopyranoside	*A. schubertii*	bulbs	[7]
126		(25*R*)-5*α*-spirostan-2*α*,3*β*,6*β*-triol 3-O-*β*-D-glucopyranosyl-(1→2)-O-[3-O-benzoyl-*β*-D-xylopyranosyl-(1→3)]-O-*β*-D-glucopyranosyl-(1→4)-*β*-D-galactopyranoside	*A. schubertii*	bulbs	[7]
127		(25*S*)-5*α*-spirostan-2*α*,3*β*,6*β*-triol 3-O-*β*-D-glucopyranosyl-(1→2)-O-[3-O-benzoyl-*β*-D-xylopyranosyl-(1→3)]-O-*β*-D-glucopyranosyl-(1→4)-*β*-D-galactopyranoside	*A. schubertii*	bulbs	[7]
128		(25*R*)-5*α*-spirostan-2*α*,3*β*,6*β*-triol 3-O-*β*-D-glucopyranosyl-(1→2)-O-[4-O-(3*S*)-3-hydroxy-3-methylglutaroyl-*β*-D-xylopyranosyl-(1→3)]-O-*β*-D-glucopyranosyl-(1→4)-*β*-D-galactopyranoside	*A. schubertii*	bulbs	[7]
			*A. giganteum*	bulbs	[6]
			*A. nigrum* L	bulbs	[43]
129		(25*S*)-5*α*-spirostan-2*α*,3*β*,6*β*-triol 3-O-*β*-D-glucopyranosyl-(1→2)-O-[4-O-(3*S*)-3-hydroxy-3-methylglutaroyl-*β*-D-xylopyranosyl-(1→3)]-O-*β*-D-glucopyranosyl-(1→4)-*β*-D-galactopyranoside	*A. schubertii*	bulbs	[7]
			*A. nigrum* L	bulbs	[43]
130		3-O-acetyl-(24*S*,25*S*)-5*α*-spirostane-2*α*,3*β*,5*α*,6*β*,24-pentol 2-O-*β*-D-glucopyranoside	*A. giganteum*	bulbs	[6]
131		methyl ester of (25*R*)-5*α*-spirostane-2*α*,3*β*,6*β*-triol 3-O-{O-*β*-D-glucopyranosyl-(1→2)-O-[4-O-(*S*)-3-hydroxy-3-methylglutaryl-*β*-D-xylopyranosyl-(1→3)]-O-*β*-D-glucopyranosyl-(1→4)-*β*-D-galactopyranoside}	*A. macleanii*	bulbs	[40]
132		tigogenin 3-O-{O-*α*-L-rhamnopyranosyl-(1→2)-O-*β*-D-xylopyranosyl-(1→2)-O-[*β*-D-xylopyranosyl-(1→3)]-*β*-D-glucopyranosyl-(1→4)-*β*-D-galactopyranoside}	*A. macleanii*	bulbs	[40]
133	macrostemonoside A	(25*R*)-5*α*-spirostan-3*β*-ol 3-O-{O-*β*-D-glucopyranosyl-(1→2)-O-[*β*-D-glucopyranosyl-(1→3)]-O-*β*-D-glucopyranosyl-(1→4)-*β*-D-galactopyraoside}	*A. chinense* G. Don	bulbs	[46]
			*A. macrostemon* Bunge	bulbs	[47]
134		(25*S*)-5*α*-spirostan-3*β*-ol 3-O-{O-*β*-D-glucopyranosyl-(1→2)-O-[*β*-D-glucopyranosyl-(1→3)]-O-*β*-D-glucopyranosyl-(1→4)-*β*-D-galactopyraoside}	*A. chinense* G. Don	bulbs	[46]
135		(25*R*)-5*α*-spirostane-2*α*,3*β*-diol 3-O-{O-*β*-D-glucopyranosyl-(1→2)-O-[*β*-D-glucopyranosyl-(1→3)]-O-*β*-D-glucopyranosyl-(1→4)-*β*-D-galactopyranoside}	*A. chinense* G. Don	bulbs	[46]
			*A. sativum* L. var. Voghiera	bulbs	[20]
136		(25*S*)-5*α*-spirostane-2*α*,3*β*-diol 3-O-{O-*β*-D-glucopyranosyl-(1→2)-O-[*β*-D-glucopyranosyl-(1→3)]-O-*β*-D-glucopyranosyl-(1→4)-*β*-D-galactopyranoside}	*A. chinense* G. Don	bulbs	[46]
137		(25*R*)-5*α*-spirostane-2*α*,3*β*-diol 3-O-{O-*β*-D-glucopyranosyl-(1→2)-O-*β*-D-glucopyranosyl-(1→4)-*β*-D-galactopyranoside}	*A. chinense* G. Don	bulbs	[46]
138		(25*S*)-5*α*-spirostane-2*α*,3*β*-diol 3-O-{O-*β*-D-glucopyranosyl-(1→2)-O-*β*-D-glucopyranosyl-(1→4)-*β*-D-galactopyranoside}	*A. chinense* G. Don	bulbs	[46]
139	agigenin	spirostan-2*α*, 3*β*, 6*β*–triol	*A. porrum* L	bulbs	[48]
				flowers	[37]
140	neoagigenin		*A. porrum* L	bulbs	[48]
			*A. minutiflorum* Regel	bulbs	[14]
141	porrigenin A	(25*R*)-5*α*-spirostan-2*β*,3*β*,6*β*-triol	*A. porrum* L	bulbs	[48]
142	neoporrigenin A	(25*S*)-5*α*-spirostan-2*β*,3*β*,6*β*-triol	*A. porrum* L	bulbs	[48]
143	yayoisaponin A	agigenin 3-O-*β*-D-glucopyranosyl-(1→3)-*β*-D-glucopyranosyl-(1→2)-[*β*-D-xylopyranosyl-(1→3)]-*β*-D-glucopyranosyl-(1→4)-*β*-D-galactopyranoside	*A. ampeloprasum* L	bulbs	[41]
144	yayoisaponin C	agigenin 3-O-*β*-D-glucopyranosyl-(1→2)-[*β*-D-glucopyranosyl-(1→3)]-*β*-D-glucopyranosyl-(1→4)-*β*-D-galactopyranoside	*A. ampeloprasum* L	bulbs	[41]
145	timosaponin A III	sarsasapogenin 3-O-*β*-D-glucopyranosyl-(1→2)-*β*-D-galactopyranoside	*A. chinense* G. Don	bulbs	[49]
146	macrostemonoside D	tigogenin 3-O-*β*-D-glucopyranosyl-(1→2)-[*β*-D-glucopyranosyl-(1→3)]-(6-acetyl-*β*-D-glucopyranosyl)-(1→4)-*β*-D-galactopyranoside	*A. chinense* G. Don	bulbs	[49]
147	neomacrostemonoside D	neotigogenin 3-O-*β*-D-glucopyranosyl-(1→2)-[*β*-D-glucopyranosyl-(1→3)]-(6-acetyl-*β*-D-glucopyranosyl)-(1→4)-*β*-D-galactopyranoside	*A. chinense* G. Don	bulbs	[49]
148		alliogenin 3-O-{O-*β*-D-glucopyranosyl-(1→2)-O-[*β*-D-xylopyranosyl-(1→3)]-O-*β*-D-glucopyranosyl-(1→4)-*β*-D-galactopyranoside}	*A. karataviense*	bulbs	[9]
149		(25*R*)-3-O-(2-hydroxybutyryl)-5*α*-spirostane-2*α*,3*β*,5,6*β*-tetrol 2-O-*β*-D-glucopyranoside	*A. karataviense*	bulbs	[9]
150		(24*S*,25*S*)-3-O-benzoyl-5*α*-spirostane-2*α*,3*β*,5,6*β*,24-pentol 2-O-*β*-D-glucopyranoside	*A. karataviense*	bulbs	[9]
151		(24*S*,25*S*)-5*α*-spirostane-2*α*,3*β*,5,6*β*,24-pentol 2,24-di-O-*β*-D-glucopyranoside	*A. karataviense*	bulbs	[9]
152		(24*S*,25*S*)-3-O-benzoyl-5*α*-spirostane-2*α*,3*β*,5,6*β*,24-pentol 2,24-di-O-*β*-D-glucopyranoside	*A. karataviense*	bulbs	[9]
153		(24*S*,25*S*)-5*α*-spirostane-2*α*,3*β*,5,6*β*,24-pentol 2-O-*β*-D-glucopyranosyl 24-O-{O-*β*-D-glucopyranosyl-(1→2)-*β*-D-glucopyranoside	*A. karataviense*	bulbs	[9]
154		(25*R*)-5*α*-spirostan-2*α*,3*β*-diol 3-O-{O-*β*-D-glucopyranosyl-(1→2)-O-[*β*-D-xylopyranosyl-(1→3)]-O-*β*-D-glucopyranosyl-(1→4)-*β*-D-galactopyranoside}	*A. porrum* L	bulbs	[50]
155		(25*R*)-5*α*-spirostan-2*α*,3*β*-diol 3-O-{O-*β*-D-glucopyranosyl-(1→3)-O-*β*-D-glucopyranosyl-(1→2)-O-[*β*-D-xylopyranosyl-(1→3)]-O-*β*-D-glucopyranosyl-(1→4)-*β*-D-galactopyranoside}	*A. porrum* L	bulbs	[50]
			*A. rotundum*	inflorescences and flower stalks	[19]
156		(25*R*)-5*α*-spirostan-3*β*,6*β*-diol 3-O-{O-*β*-D-glucopyranosyl-(1→2)-O-[*β*-D-xylopyranosyl-(1→3)]-O-*β*-D-glucopyranosyl-(1→4)-*β*-D-galactopyranoside}	*A. porrum* L	bulbs	[50]
			*A. leucanthum*	flowers	[42]
157		(25*R*)-5*α*-spirostan-3*β*,6*β*-diol 3-O-{O-*β*-D-glucopyranosyl-(1→3)-O-*β*-D-glucopyranosyl-(1→2)-O-[*β*-D-xylopyranosyl-(1→3)]-O-*β*-D-glucopyranosyl-(1→4)-*β*-D-galactopyranoside}	*A. porrum* L	bulbs	[50]
158	tuberoside D	(25*S*)-5*α*-spirostane-2*α*,3*β*‚-diol 3-O-*α*-L-rhamnopyranosyl-(1→2)-O-[*α*-L-rhamnopyranosyl-(1→4)]-O-*β*-D-glucopyranoside	*A. tuberosum*	seeds	[51]
159	tuberoside E	(25*S*)-5*α*-spirostan-2*α*,3*β*-diol 3-O-*β*-D-glucopyranosyl-(1→2)-O-[*α*-L-rhamnopyranosyl-(1→4)]O-*β*-D-glucopyranoside	*A. tuberosum*	seeds	[51]
160		(25*S*)-spirostane-3*β*,5*β*,6*α*-triol 3-O-*α*-L-rhamnopyranosyl-(1→4)-*β*-D-glucopyranoside	*A. tuberosum*	seeds	[52]
161		(25*S*)-5*β*-spirostane-3*β*,6*α*-diol (25epi-ruizgenin) 3-O-*α*-L-rhamnopyranosyl-(1→4)-*β*-D-glucopyranoside	*A. tuberosum*	seeds	[52]
162	tuberoside J	(25*R*)-5*α*-spirostan-2*α*,3*β*,27-triol 3-O-*α*-L-rhamnopyranosyl-(1→2)-*β*-D-glucopyranoside	*A. tuberosum*	seeds	[53]
163	tuberoside K	(25*R*)-5*α*-spirostan-2*α*,3*β*,27-triol 3-O-*α*-L-rhamnopyranosyl-(1→2)-[*α*-L-rhamnopyranosyl-(1→4)]-*β*-D-glucopyranoside	*A. tuberosum*	seeds	[53]
164	tuberoside L	27-O-*β*-D-glucopyranosyl-(25*R*)-5*α*-spirostan-2*α*,3*β*,27-triol 3-O-*α*-D-rhamnopyranosyl-(1→2)-[*α*-L-rhamnopyranosyl-(1→4)]-*β*-D-glucopyranoside	*A. tuberosum*	seeds	[53]
165	tuberoside	(2*α*,3*β*,5*α*,25*S*)-2,3,27-trihydroxyspirostane 3-O-*α*-L-rhamnopyranoyl-(1→2)-O-[*α*-L-rhamnopyranoyl-(1→4)]-*β*-D-glucopyranoside	*A. tuberosum* Rottl. ex Spreng	seeds	[54]
166	tuberoside N	(25*S*)-5*β*-spirostan-2*β*,3*β*-diol 3-O-*β*-D-glucopyranosyl-(1→2)-[*α*-L-rhamnopyranosyl (1→4)]-*β*-D-glucopyranoside	*A. tuberosum* L	seeds	[29]
167	tuberoside O	(25*S*)-5*β*-spirostan-2*β*,3*β*,5-triol 3-O-*β*-D-glucopyranoside	*A. tuberosum* L	seeds	[29]
				roots	[55]
168	tuberoside P	(25*S*)-5*β*-spirostan-2*β*,3*β*, 5-triol 3-O-*α*-L-rhamnopyranosyl (1→4)-*β*-D-glucopyranoside	*A. tuberosum* L	seeds	[29]
169	tuberoside Q	(24*S*,25*S*)-5*β*-spirostan-2*β*,3*β*,5,24-tetraol 3-O-*α*-L-rhamnopyranosyl (1→4)-*β*-D-glucopyranoside	*A. tuberosum* L	seeds	[29]
170	agapanthagenin		*A. elburzense*	bulbs	[10]
171	hirtifolioside D	spirostan-2*α*,3*β*,6*β*-triol 3-O-*β*-D-xylopyranosyl-(1→3)-O-*β*-D-glucopyranosyl-(1→4)-O-*β*-D-galactopyranoside	*A. hirtifolium* Boiss	flowers	[12]
172		agapanthagenin 3-O-*β*-D-glucopyranoside	*A. hirtifolium* Boiss	flowers	[12]
173	atroviolacegenin	(25*R*)-5*α*-spirostan-2*α*,3*β*,6*β*,27-tetrol	*A. atroviolaceum*	flowers	[56]
174	atroviolaceoside	(25*R*)-5*α*-spirostan-2*α*,3*β*,6*β*,27-tetrol 3-O-*β*-D-glucopyranosyl-(1→4)-O-*β*-D-galactopyranoside	*A. atroviolaceum*	flowers	[56]
175	minutoside B	(25*S*)-spirostan-2*α*,3*β*,6*β*-triol 3-O-*β*-D-xylopyranosyl-(1→3)-O-*β*-D-glucopyranosyl-(1→4)-O-*β*-D-galactopyranoside	*A. minutiflorum* Regel	bulbs	[14]
176	eruboside B	*β*-chlorogenin 3-O-*β*-glucopyranosyl(1→2)-[*β*-glucopyranosyl-(1→3)]-*β*-glucopyranosyl(1→4)-*β*-galactopyranoside	*A. leucanthum*	flowers	[42]
			*A. sativum* L	bulbs	[2]
177	leucospiroside A	(25*R*)-5*α*-spirostane-2*α*,3*β*,6*β*-triol 3-O-*β*-glucopyranosyl-(1→3)-*β*-glucopyranosyl-(1→2)-[*β*-glucopyranosyl-(1→3)]-*β*-glucopyranosyl-(1→4)-*β*-galactopyranoside	*A. leucanthum*	flowers	[42]
			*A. ampeloprasum* var. *porrum*	bulbs	[57]
178		(25*R*)-5*α*-spirostane-2*α*,3*β*,6*β*-triol 3-O-*β*-D-glucopyranosyl-(1→2)-*β*-D-glucopyranosyl-(1→4)-*β*-D-galactopyranoside	*A. leucanthum*	flowers	[42]
179		(25*R*)-5*α*-spirostane-3*β*,6*β*-diol 3-O-*β*-D-glucopyranosyl-(1→3)-*β*-D-glucopyranosyl-(1→2)-[*β*-D-glucopyranosyl-(1→3)]-*β*-D-glucopyranosyl(1→4)-*β*-D-galactopyranoside	*A. leucanthum*	flowers	[42]
180	tuberoside A	(24*S*, 25*S*)-5*β*-spirostan-2*β*,3*β*,24-triol 3-O-a-L-rhamnopyranoyl-(1→2)-O-[*α*-L-rhamnopyranosyl-(1→4)]-*β*-D-glucopyranoside	*A. tuberosum* Rottl. ex Spreng	seeds	[58]
181		(3*β*,5*α*,6*β*,25*R*)-6-[(*β*-D-glucopyranosyl)oxy]spirostan-3-yl O-*β*-D-glucopyranosyl-(1→2)-O-[*β*-D-glucopyranosyl-(1→3)]-*β*-D-galactopyranoside	*A. ampeloprasum* var. *porrum*	bulbs	[59]
182	nigroside A1	25(*R*)-5*α*-spirostan-2*α*,3*β*,6*β*-triol 3-O-*α*-L-rhamnopyranosyl-(1→2)-*β*-D-glucopyranoside	*A. nigrum* L	bulbs	[43]
183	nigroside A2	25(*S*)-5*α*-spirostan-2*α*,3*β*,6*β*-triol 3-O-*α*-L-rhamnopyranosyl-(1→2)-*β*-D-glucopyranoside	*A. nigrum* L	bulbs	[43]
184	nigroside B1	25(*R*)-5*α*-spirostan-2*α*,3*β*,6*β*-trio 1-2-O-[*β*-D-glucopyranosyl]-3-O-*β*-D-galactopyranoside	*A. nigrum* L	bulbs	[43]
185	nigroside B2	25(*S*)-5*α*-spirostan-2*α*,3*β*,6*β*-trio 1-2-O-[*β*-D-glucopyranosyl]-3-O-*β*-D-galactopyranoside	*A. nigrum* L	bulbs	[43]
186		*β*-D-glucopyranosyl-(1→2)-[4-O-(3-hydroxy-3-methylglutaryl)-*β*-D-xylopyranosyl-(1→3)]-*β*-D-glucopyranosyl-(1→4)-*β*-D-galactopyranosyl-(1→3)-(25*R*)-5*α*-spirostan-2*α*,3*β*-diol	*A. cyrillii*	bulbs	[38]
187	persicoside A	(25*S*)-spirostan-2*α*,3*β*,6*β*-triol 3-O-[*β*-D-glucopyranosyl-(1→3)] [*β*-D-xylopyranosyl-(1→2)]-*β*-D-glucopyranosyl-(1→4)-*β*-D-galactopyranoside	*A. ampeloprasum* subsp. *persicum*	seeds	[21]
188	persicoside B	(25*S*)-spirostan-2*α*,3*β*,6*β*-triol 3-O-[*β*-D-xylopyranosyl-(1→3)] [*α*-L-rhamnopyranosyl(1→2)]-*β*-D-glucopyranosyl-(1→4)-O-*β*-D-galactopyranoside	*A. ampeloprasum* subsp. *persicum*	seeds	[21]
189		(25*R*)-5*α*-spirostan-3*β*,11a*α*-diol 3-O-*β*-D-glucopyranosyl-(1→3)-[*β*-D-glucopyranosyl-(1→4)]-*β*-D-galactopyranoside	*A. schoenoprasum*	whole plants	[24]
190	tuberoside B	(24*S*,25*S*)-5*β*-spirostan-2*α*,3*β*,5,24-tetraol 3-O-*α*-L-rhamnopyranoyl-(1→2)-O-[*α*-L-rhamnopyranosyl-(1→4)]-*β*-D-glucopyranoside	*A. tuberosum* Rottl. ex Spreng	seeds	[60]
191	tuberosine A	(25*S*)-5*β*-spirostan-2*β*,3*β*-diol 3-O-*β*-D-glucopyranoside	*A. tuberosum*	roots	[55]
192	tuberosine B	(25*S*)-5*β*-spirostan-2*β*,3*β*,19-triol 3-O-*β*-D-glucopyranoside	*A. tuberosum*	roots	[55]
193	tuberosine C	(25*S*)-5*β*-spirostan-2*β*,3*β*-diol 3-O-*α*-L-rhamnopyranoyl-(1→4)-O-*β*-D-glucopyranoside	*A. tuberosum*	roots	[55]
194	25(S)-schidigera-saponin D5		*A. tuberosum*	roots	[55]
195	shatavarin IV		*A. tuberosum*	roots	[55]
196	karatavioside I	(24*S*,25*S*)-24-[(O-*β*-D-glucopyranosyl(1→2)-*β*-D-glucopyranosyl)oxy]-2*α*,5*α*,6*β*-trihydroxyspirostan-3*β*-yl O-*β*-D-glucopyranosyl-(1→2)-[*β*-D-xylopyranosyl-(1→3)]-*β*-D-glucopyranosyl-(1→4)-*β*-D-galactopyranoside	*A. karataviense*	bulbs	[25]
197	neogitogenin		*A. chinense* G. Don	bulbs	[61]
198		(25*R*)-5*α*-spirostan-3*β*-yl-3-O-acetyl-O-*β*-D-glucopyranosyl-(1→2)-O-[*β*-D-glucopyranosyl-(1→3)]-O-*β*-D-glucopyranosyl-(1→4)-*β*-D-galactopyranoside	*A. chinense* G. Don	bulbs	[61]
199		(25*S*)-5*α*-spirostane-3*β*-ol-3-O-{O-*β*-D-glucopyranosyl-(1→2)-O-*β*-D-glucopyranosyl-(1→4)-*β*-D-galactopyranoside}	*A. chinense* G. Don	bulbs	[61]
200	allimacroside B	(24*S*,25*S*)-24-[(*β*-D-glucopyranosyl)oxy]-5*α*-spirostan-3*β*-yl-O-*β*-D-glucopyranosyl(1→2)-[*β*-D-glucopyranosyl(1→3)]-*β*-D-glucopyranosyl(1→4)-*β*-D-galactopyranoside	*A. macrostemon* Bunge	whole plants	[26]
201	yayoisaponin A/alliporin	(2*α*, 3*β*, 6*β*, 25*R*)-2,6-dihydroxyspirostan-3-yl *β*-D-glucopyranosyl-(1→3)-*β*-D-glucopranosyl-(1→2)-[*β*-D-xylopyranosyl-(1→3)]-*β*-D-glucopyranosyl]-(1→4)-*β*-D-galactopyranoside	*A. porrum* L	flowers	[37]
202	alliospiroside A	(25*S*)-3*β*-hydroxyspirost-5-en-1*β*-yl-2-O-(6-deoxy-*α*-L-mannopyranosyl)-*α*-L-arabinopyranoside	*A. cepa* L	collective fruit	[62]
			*A. cepa* L. Aggregatum group	roots	[63]
203	alliospiroside B		*A. cepa* L	collective fruit	[62]
204	alliospiroside D		*A. cepa* L	collective fruit	[62]
205		(25*R*)-spirost-5-en-3*β*-ol (diosgenin) 3-O-{O-*α*-L-rhamnopyranosyl-(1→2)-O-[O-*α*-L-rhamnopyranosyl-(1→4)-*α*-L-rhamnopyranosyl-(1→4)]-*β*-D-glucopyanoside}	*A. senescens*	bulbs	[40]
206		diosgenin 3-O-{O-*α*-L-rhamnopyranosyl-(1→2)-O-[*β*-D-glucopyranosyl-(1→3)]-*β*-D-glucopyranoside}	*A. senescens*	bulbs	[40]
207	dioscin		*A. ampeloprasum* L	bulbs	[41]
208		(25*R*)-spirost-5-ene-2*α*,3*β*-diol 3-O-{O-*β*-D-glucopyranosyl-(1→2)-O-[*β*-D-xylopyranosyl-(1→3]-O-*β*-D-glucopyranosyl-(1→4)-*β*-D-galactopyranoside}	*A. karataviense*	bulbs	[9]
209	β-chacotriosyl lilagenin		*A. tuberosum*	seeds	[52]
210		(20*S*,25*S*)-spirost-5-en-3*β*,12*β*,21-triol 3-O-*α*-L-rhamnopyranosyl-(1→2)-*β*-D-glucopyranoside	*A. schoenoprasum*	whole plants	[24]
211		(20*S*,25*S*)-spirost-5-en-3*β*,11*α*,21-triol 3-O-*α*-L-rhamnopyranosyl-(1→2)-*β*-D-glucopyranoside	*A. schoenoprasum*	whole plants	[24]
212		diosgenin 3-O-*α*-L-rhamnopyranosyl-(1→2)-O-*β*-D-glucopyranoside	*A. schoenoprasum*	whole plants	[24]
213	deltonin	diosgenin 3-O-*β*-D-glucopyranosyl-(1→ 4)-[*α*-L-rhamnopyranosyl-(1→2)]-*β*-D-glucopyranoside	*A. schoenoprasum*	whole plants	[24]
214	karatavioside H	(24*S*,25*S*)-24-[(O-*β*-D-glucopyranosyl-(1→2)-*β*-D-glucopyranosyl) oxy]-2*α*-hydroxyspirost-5-en-3*β*-yl O-*β*-D-glucopyranosyl-(1→2)-[*β*-D-xylopyranosyl-(1→3)]-*β*-D-glucopyranosyl-(1→4)-*β*-D-galactopyranoside	*A. karataviense*	bulbs	[25]
215	allimacroside C	(24*S*,25*S*)-24-[(*β*-D-glucopyranosyl)oxy]-spirost-5-ene-3*β*,24-diol-3-O-*β*-D-glucopyranosyl(1→2)-[*β*-D-glucopyranosyl(1→3)]-*β*-D-glucopyranosyl(1→4)-*β*-D-galactopyranoside	*A. macrostemon* Bunge	whole plants	[26]
216		5*α*-spirostane 25(27)-ene-2*α*,3*β*-diol-3-O-{O-*β*-D-glucopyranosyl-(1→2)-O-*β*-D-glucopyranosyl-(1→4)-*β*-D-galactopyranoside}	*A. chinense* G. Don	bulbs	[61]
217	porrigenin B	(25*R*)-2-oxo-5*α*-spirostan-3*β*,6*β*-diol	*A. porrum* L	bulbs	[48]
218	neoporrigenin B	(25*S*)-2-oxo-5*α*-spirostan-3*β*,6*β*-diol	*A. porrum* L	bulbs	[48]
219	yayoisaponin B	porrigenin B 3-O-*β*-D-glucopyranosyl-(1→3)-*β*-D-glucopyranosyl-(1→2)-[*β*-D-xylopyranosyl-(1→3)]-*β*-D-glucopyranosyl-(1→4)-*β*-D-galactopyranoside	*A. ampeloprasum* L	bulbs	[41]
220		(3*β*,5*α*,6*β*,25*R*)-3-{(O-*β*-D-glucopyranosyl-(1→3)-*β*-D-glucopyranosyl-(1→2)-O-[O-*β*-D-glucopyranosyl-(1→3)]-O-*β*-D-glucopyranosyl-(1→4)-*β*-D-galactopyranosyl)oxy}-6-hydroxyspirostan-2-one	*A. ampeloprasum* var. *porrum*	bulbs	[64]
221	3-keto umbilicagenin A	(25*R*)-3-keto-spirostan-2*α*,5*α*,6*β*-triol	*A. umbilicatum* Boiss	flowers	[65]
222	3-keto umbilicagenin B	(25*R*)-3-keto-spirostan-2*α*,5*α*-diol	*A. umbilicatum* Boiss	flowers	[65]
223	anzurogenin A	2*α*,3*β*,5*β*-trihydroxy-(25*R*)-spirostan-6-one	*A. suvorovii* and *A. stipitatum*	fruit	[66]
224	anzuroside	(24*S*, 25*S*)-2*α*,3*β*,5,24-tetrahydroxy-5*β*-spirostan-6-one 24-O-*β*-D-glucopyranoside	*A. suvorovii* and *A. stipitatum*	fruits	[67]
225	anzurogenin C	(24*S*, 25*S*)-2*α*,3*β*,5,24-tetrahydroxy-5*β*-spirostan-6-one	*A. suvorovii* and *A. stipitatum*	fruits	[67]
			*A. chinense* G. Don	bulbs	[49]
226		(25*R*)-3*β*-hydroxy-5*α*-spirostan-6-one (laxogenin) 3-O-{O-*α*-L-arabinopyranosyl-(1→6)-*β*-D-glucopyranoside}	*A. chinense* G. Don	bulbs	[46]
227		laxogenin 3-O-{O-(2-O-acetyl-*α*-L-arabinopyranosyl)-(1→6)-*β*-D-glucopyranoside}	*A. chinense* G. Don	bulbs	[46]
228	xiebai-saponin I	laxogenin 3-O-{O-*β*-D-xylopyranosyl-(1→4)-O-[*α*-L-arabinopyranosyl-(1→6)]-*β*-D-glucopyranoside}	*A. chinense* G. Don	bulbs	[46]
229	chinenosideVI	(25*R*)-24-O-*β*-D-glucopyranosyl-3*β*,24*β*-dihydroxy-5*α*-spirost 3-O-*α*-arabinopyranosyl-(1→6)-*β*-D-glucopyranoside	*A. chinense* G. Don	bulbs	[49]
230		laxogenin 3-O-*β*-D-glucopyranosyl-(1→4)-[*α*-L-arabinopyranosyl-(1→6)]-*β*-D-glucopyranoside	*A. chinense* G. Don	bulbs	[49]
231	laxogenin		*A. chinense* G. Don	bulbs	[68]
232		laxogenin 3-O-*α*-L-rhamnopyranosyl-(1→2)-[*β*-D-glucopyranosyl-(1→4)]-*β*-D-glucopyranoside	*A. schoenoprasum*	whole plants	[24]
233		laxogenin 3-O-*α*-L-rhamnopyranosyl-(1→2)-*β*-D-glucopyranoside	*A. schoenoprasum*	whole plants	[24]
234		laxogenin 3-O-*β*-D-glucopyranoside	*A. chinense* G. Don	bulbs	[61]
235		laxogenin 3-O-{*β*-D-xylopyranosyl-(1→4)-*β*-D-glucopyranoside}	*A. chinense* G. Don	bulbs	[61]
236		(25*R*)-5*α*-spirostan 3-O-{O-(4-O-acetyl-*α*-L-arabinopyranosyl)-(1→6)-*β*-D-glucopyranoside}	*A. chinense* G. Don	bulbs	[61]
237		(25*R*)-3*β*-hydroxy-5*β*-spirostan-6-one 3-O-*β*-D-xylopyranosyl(1→4)-[*α*-L-arabinopyranosyl-(1→6)]-*β*-D-glucopyranoside	*A. chinense* G. Don	bulbs	[61]
238		(25*R*)-3*β*-hydroxy-5*α*-spirostan-6-one 3-O-{[O-*β*-D-glucopyranosyl-(1→3)-O-*β*-D-xylopyranosyl]-(1→4)-O-[*α*-L-arabinopyranosyl-(1→6)]}-*β*-D-glucopyranoside	*A. chinense* G. Don	bulbs	[61]
239		(25*S*)-3*β*,24*β*-dihydroxy-5*α*-spirostan-6-one 3-O-[*α*-L-arabinopyranosyl-(1→6)]-*β*-D-glucopyranoside	*A. chinense* G. Don	bulbs	[61]
240		(25*S*)-24-O-*β*-D-glucopyranosyl-3*β*,24*β*-dihydroxy-5*α*-spirostan-6-one	*A. chinense* G. Don	bulbs	[61]
241	12-keto-porrigenin	(25*R*)-5*α*-spirostan-3*β*, 6*β*-diol-12-one	*A. porrum* L		[69]
242		(25*S*)-5*α*-spirostan-3*β*, 6*β*-diol-12-one	*A. porrum* L		[69]
243		(25*R*)-3*β*,6*β*‚dihydroxy-5*α*-spirostan-12-one-3-O-{O-*β*-D-glucopyranosyl-(1→2)-O-[*β*-D-xylopyranosyl-(1→3)]-O-*β*-D-glucopyranosyl-(1→4)-*β*-D-galactopyranoside}	*A. porrum* L	bulbs	[36]
244		(25*R*)-5*α*-spirostan-3*β*,6*β*-diol-12-one 3-O-*β*-D-glucopyranosyl-(1→2)-[*β*-D-fucopyranosyl-(1→3)]-*β*-D-glucopyranosyl-(1→4)-*β*-D-galactopyranoside	*A. porrum* L	bulbs	[70]
245	porrigenin C		*A. porrum* L		[71]
246	neoporrigenin C		*A. porrum* L		[71]
247		(25*R*)-3*β*,6*β*-dihydroxy-5*α*-spirostan-2,12-dione-3-O-{O-*β*-D-glucopyranosyl-(1→2)-O-[*β*-D-xylopyranosyl-(1→3)]-O-*β*-D-glucopyranosyl-(1→4)-*β*-D-galactopyranoside}	*A. porrum* L	bulbs	[36]
248		(25*R*)-5*α*-spirostane-3*β*,6*β*-diol-2,12-dione 3-O-{*β*-D-glucopyranosyl-(1→3)-*β*-D-glucopyranosyl-(1→2)-[*β* -D-xylopyranosyl-(1→3)]-*β*-D-glucopyranosyl-(1→4)-*β*-D-galactopyranoside}	*A. porrum* L	bulbs	[72]
249		(25*R*)-spirost-4-ene-3-one-2-ol	*A. fistulosum* L	seeds	[73]
250		(25*R*)-spirost-1,4-diene-3-one-2,6-diol	*A. fistulosum* L	seeds	[73]
251		(25*R*)-spirost-1,4-diene-3-one-2-ol	*A. fistulosum* L	seeds	[73]
252		(25*R*)-19-norspirosta-1,3,5 (10)-triene-4-methyl-2-ol	*A. fistulosum* L	seeds	[73]
253	anzurogenin B	2*α*,5*α*-epoxy-(25*R*)-spirostan-3*β*,6*β*-diol	*A. suvorovii* and *A. stipitatum*	fruit	[74]
254		(22*S*)-cholest-5-ene-1*β*,3*β*,16*β*,22-tetraol 1-O-*α*-L-rhamnopyranoside 16-O-{O-*α*-L-rhamnopyranosyl-(1→3)-*β*-D-glucopyranoside}	*A. albopilosum*	bulbs	[5]
255		(22*S*)-cholest-5-ene-1*β*,3*β*,16*β*,22-tetraol 16-O-{O-*β*-D-glucopyranosyl-(1→3)-*β*-D-glucopyranoside}	*A. ostrowskianum*	bulbs	[5]
256		(22*S*)-cholest-5-ene-1*β*,3*β*,16*β*,22-tetrol 1,3-di-O,O’-*α*-L-rhamnopyranoside 16-O-*β*-D-glucopyranoside	*A. macleanii*	bulbs	[40]
257		(22*S*)-cholest-5-ene-1*β*,3*β*,16*β*,22-tetrol 1,16-di-O-*β*-D-glucopyranoside	*A. jesdianum*	bulbs	[35]
258		(22*S*)-cholest-5-ene-1*β*,3*β*,16*β*,22-tetrol 1-O-*α*-L-rhamnopyranosyl 16-O-*β*-D-glucopyranoside	*A. jesdianum*	bulbs	[35]
259		22*S*-cholest-5-ene-1*β*,3*β*,16*β*,22-tetrol 1-O-*α*-L-rhamnopyranosyl 16-O-*β*-D-galactopyranoside	*A. porrum* L	bulbs	[36]
260		22*S*-cholest-5-ene-1*β*,3*β*,16*β*,22-tetrol 1-O-[O-*β*-D-glucopyranosyl-(1→4)-*α*-L-rhamnopyranoside] 16-O-*β*-D-galactopyranoside	*A. porrum* L	bulbs	[36]
261	tuberoside U	16-O-*β*-D-glucopyranosyl-(22*S*,25*S*)-cholest-5-ene-3*β*,16*β*, 22, 26-tetraol 3-O-*α*-L-rhamnopyranosyl (1→2)-[*α*-L-rhamnopyranosyl (1→4)]-*β*-D-glucopyranoside	*A. tuberosum* L	seeds	[29]
262	nigroside C	(22*S*)-cholest-5-ene-1*β*,3*β*,16*β*, 22-tetraol 1-O-[*α*-L-rhamnopyranosyl] 16-O-*α*-L-rhamnopyranosyl-(1→3)-*β*-D-galactopyranoside	*A. nigrum* L	bulbs	[43]
263	nigroside D	(22*S*)-cholest-5-ene-1*β*,3*β*,16*β*,22-tetraol 16-O-*α*-L-rhamnopyranosyl-(1→3)-*β*-D-galactopyranoside	*A. nigrum* L	bulbs	[43]
264	persicoside E	(22*S*)-cholesta-1*β*,3*β*,16*β*,22*β*-tetraol 5-en 1-O-*α*-L-rhamnopyranosyl 16-O-*α*-L-rhamnopyranosyl (1→2)-*β*-D-galactopyranoside	*A. ampeloprasum* subsp. *persicum*	seeds	[21]
265	karatavioside J	(22*S*)-16*β*-[(*β*-D-glucopyranosyl)oxy]-22-hydroxycholest-5-en-3*β*-yl O-*β*-D-glucopyranosyl-(1→2)-[*β*-D-xylopyranosyl-(1→3)]-*β*-D-glucopyranosyl-(1→ 4)-*β*-D-galactopyranoside	*A. karataviense*	bulbs	[25]
266	karatavioside K	(22*S*)-16*β*-[(O-*β*-D-glucopyranosyl-(1→3)-*β*-D-glucopyranosyl)oxy]-22-hydroxycholest-5-en-3*β*-yl O-*β*-D-glucopyranosyl-(1→2)-[*β*-D-xylopyranosyl-(1→3)]*β*-D-glucopyranosyl-(1→4)-*β*-D-galactopyranoside	*A. karataviense*	bulbs	[25]
267		1*β*,3*β*,16*β*-trihydroxy-5*α*-cholestan-22-one 1-O-*α*-L-rhamnopyranoside 16-O-{O-*α*-L-rhamnopyranosyl-(1→3)-*β*-D-glucopyranoside}	*A. albopilosum*	bulbs	[5]
268		1*β*,3*β*,16*β*-trihydroxycholest-5-en-22-one 1-O-*α*-L-rhamnopyranoside 16-O-{O-*α*-L-rhamnopyranosyl-(1→3)-*β*-D-glucopyranoside}	*A. albopilosum*	bulbs	[5]
269	2,3-seco-porrigenin	(25*R*)-5*α*-2,3-secospirostan-2,3-dioic acid-6*β*-hydroxy-3,6-γ-lactone	*A. porrum* L		[69]
270		(25*S*)-5*α*-2,3-secospirostan-2,3-dioic acid-6*β*-hydroxy-3,6-γ-lactone	*A. porrum* L		[69]
271		3-O-*α*-L-rhamnopyranosyl-(1→4)-*β*-D-glucopyranosyl 3*β*,5*β*,6*α*,16*β*-tetrahydroxypregnane 16-(5-O-*β*-D-glucopyranoyl-4(*S*)-methyl-5-hydroxypentanoic acid) ester	*A. tuberosum* Rottler	seeds	[11]
272		5*α*-cholano-22,16-lactone-3-O-*β*-D-glucopyranosyl-(1→2)-[*β*-D-glucopyranosyl-(1→3)]-*β*-D-glucopyranosyl-(1→4)-*β*-D-galacopyranoside	*A. chinense* G. Don	bulbs	[13]
273		6-ketone-5*α*-cholano-22,16-lactone-3-O-*β*-D-6-xylopyranosyl-(1→4)-[*α*-L-arabinopyranosyl-(1→6)]-*β*-D-glucopyranoside	*A. chinense* G. Don	bulbs	[13]
274	allimacroside A	pregna-5,16-dien-3*β*-ol-20-one-3-O-*β*-D-glucopyranosyl(1→2)-[*β*-D-glucopyranosyl(1→3)]-*β*-Dglucopyranosyl(1→4)-*β*-D-galactopyranoside	*A. macrostemon* Bunge	whole plants	[26]

## Data Availability

Not applicable.
